# Ethnomedicinal knowledge of the rural communities of Dhirkot, Azad Jammu and Kashmir, Pakistan

**DOI:** 10.1186/s13002-019-0323-2

**Published:** 2019-08-30

**Authors:** Asia Farooq, Muhammad Shoaib Amjad, Khalid Ahmad, Muhammad Altaf, Muhammad Umair, Arshad Mehmood Abbasi

**Affiliations:** 1 Department of Botany, Women University of Azad Jammu & Kashmir, Bagh, Pakistan; 2 Department of Environment Sciences, COMSATS University Islamabad, Abbottabad Campus, 22060 Pakistan; 3 Department of Zoology, Women University of Azad Jammu & Kashmir, Bagh, Pakistan; 4 School of Agriculture and Biology, Shanghai Jiao Tong University, Shanghai, 200240 China

**Keywords:** Traditional knowledge, Medicinal plants, FC, ICF, Dhirkot

## Abstract

**Background:**

Being an isolated locality and having a tough mountainous terrain, strong ethnomedicinal practices still prevail in Dhirkot and its allied areas, which have been rarely explored yet. The present study was intended with the aim to document and compare the traditional knowledge of local communities on botanical taxa of Dhirkot, Azad Jammu, and Kashmir.

**Methodology:**

Ethnomedicinal data were collected from 74 informants using a semi-structured questionnaire in addition to field observation and group discussion. Various indices were also used to evaluate the ethnomedicinal data. Furthermore, the present findings were compared with previous reports to assess data novelty.

**Result:**

A total of 140 medicinal plant species belonging to 55 families were recorded, which are used by local communities to treat 12 disease categories. Asteraceae was dominating with 20 species, followed by Poaceae, Lamiaceae, and Rosaceae (14, 11, and 10 species, respectively). Herbs were leading with 66% contribution, whereas leaves were the most utilized plant part with 29% utilization and decoction was the common mode of administration. *Viola canescens* depicted the highest use value and relative frequency of citation (1.7 and 0.92, respectively). Maximum informant consensus factor (0.88) was calculated for digestive and liver disorders. Five plant species including *Berberis lycium Mentha arvensis Pyrus malus*, *Taraxacum officinale*, and *Viola canescens* had 100% fidelity level.

**Conclusion:**

Dhirkot and its allied areas harbor rich botanical and cultural diversity because of its unique geography and diverse climatic conditions. However, mostly, traditional ethnobotanical knowledge is restricted to healers, midwives, and older people, and could be extinct in the near future. Therefore, such documentation not only conserves traditional knowledge but may also contribute significantly to novel drug resources.

## Background

Medicinal plants are an important element of aboriginal curative systems. This knowledge is considered as a part of cultural assets [1] However, many indigenous groups fail to sustain and preserve this communal knowledge [2] that is why the systematic evaluation of this knowledge in order to contribute to health care in marginalized areas has been sighted in programs of national and international organizations [3]. In developing countries, most of the local communities are still relying on plant-based medicines [4]. The use of medicinal plants is a valuable source of income for poor communities but knowledge on therapeutic plants is decreasing gradually due to the progression in the present health care system and rapid urbanization [5, 6]. Therefore, such rich tradition should be preserved through a reliable approach before it gets lost due to various anthropogenic and other causes.

There is an amazing growing interest in the alternative systems of therapeutics on a global level [7]. The factors contributing towards the potential use of herbal drugs in developing countries are accessibility, affordability, and historical and cultural background besides a holistic approach to health problems, safety, lack of adverse reaction, and side effects [8, 9]. The use of plants as medicine ranges from 4 to 20% in different countries and about 2500 species are traded internationally. Pakistan has about 6000 species of higher plants, and among them, 10–30% of the flora is used for medicinal purposes in various areas [10, 11]. The tradition of using medicinal plants in Pakistan for the treatment of various ailments is very mature, based predominantly on the Unani system of medicine. This traditional medicine sector has become an important source of health care, especially in rural and tribal areas of the country where it is considered as first-line treatment [12].

Azad Jammu and Kashmir (AJ&K) is characterized by its diverse habitats, climate, and soil [13–16]. It is located in North-East of Pakistan and is stuffed with natural resources particularly plant flora [17]. AJK has a wide range of mountainous ecosystems which are affluent in fauna and flora. Due to extraordinary climatic conditions, the area has three vegetation groups (deserts, alpine, and grasslands). Natural and anthropogenic stresses have a great effect on the natural environment and ecosystems of the area [18]. Previously, different researchers reported ethnomedicinal uses of plant species from other parts of AJ&K [16, 19, 20]. However, the present research area is rarely reported except in one study, which was conducted about 16 years ago [21]. We hypothesize that older people are more familiar with ethnomedicinal uses of plant species compared to younger people and formal education is not predictive of the traditional knowledge level of indigenous people. Moreover, among the local communities, having the same culture usage or importance of a plant species may vary. Therefore, the present study was designed to document the traditional knowledge of plant species and its quantitative assessment and to associate the frequency of occurrence with ethnomedicinal uses of plant species.

## Materials and methods

### Study area

**Fig. 1:**
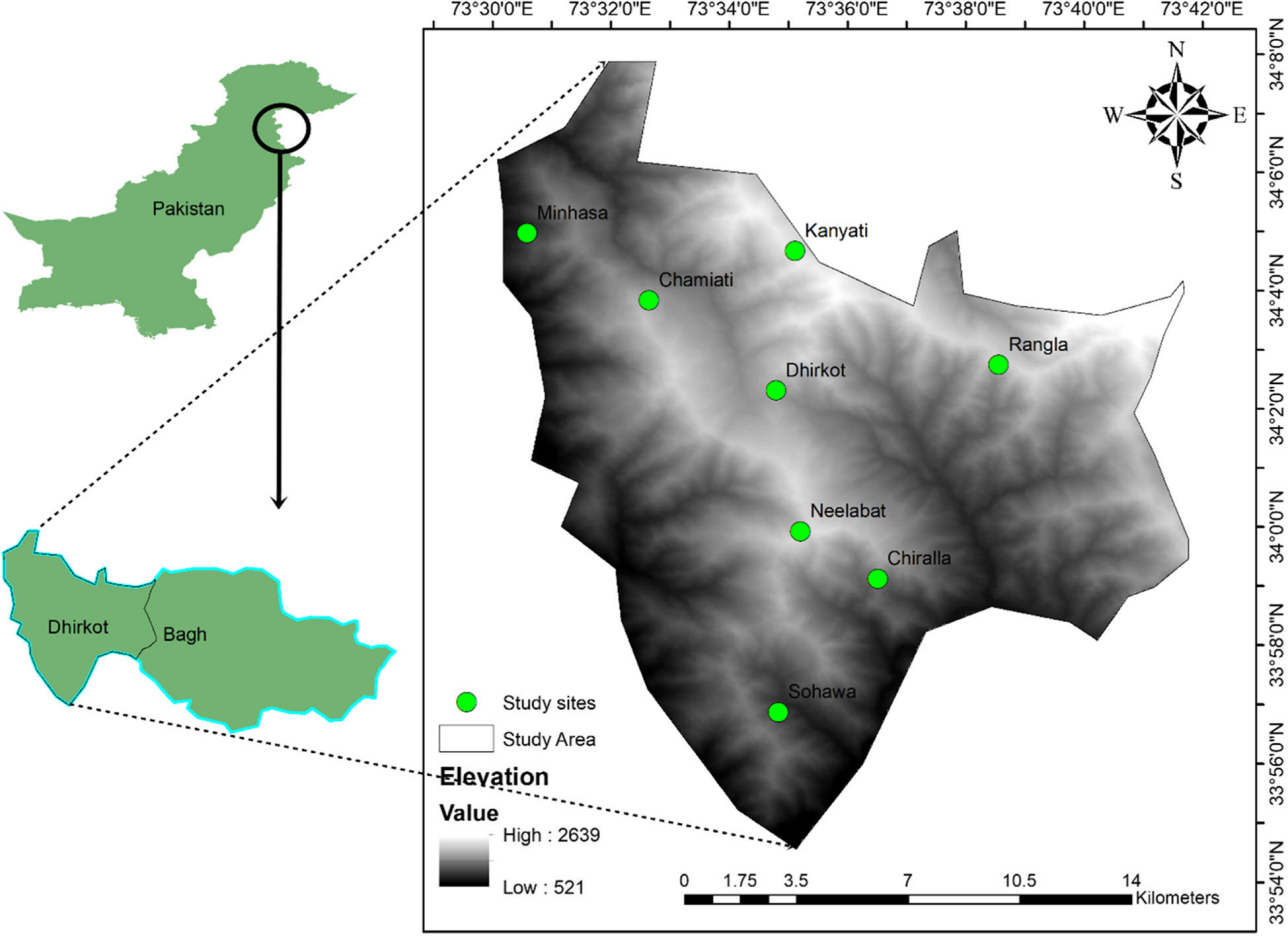
Map of the study area

**Fig. 2:**
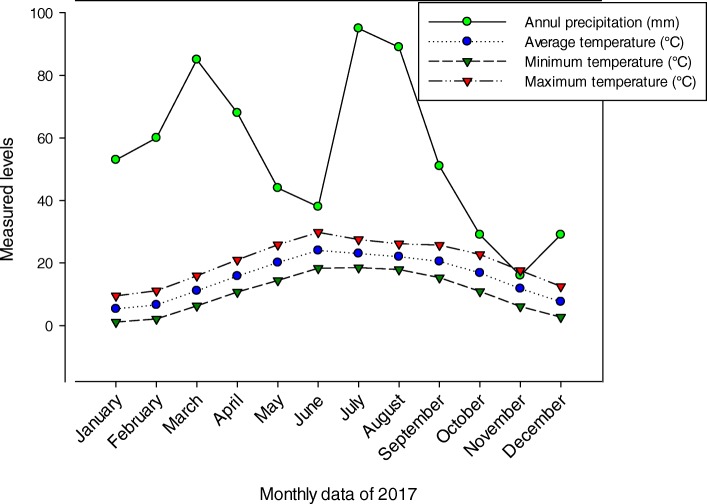
Precipitation and temperature data of the study area for the year 2017

Dhirkot is a diversity-rich mountainous area of district Bagh, Azad Jammu, & Kashmir, Pakistan. It is situated 55 km southeast of Muzaffarabad (the capital city for Azad Jammu and Kashmir) and 132 km from Islamabad. It is located on latitude 33° 57′ N and longitude 73° 36′ E (Fig. 1), covering an area of 150 km square with an altitudinal variation of 850–2200 m [22]. The climate of the study area is of a subtropical humid and moist temperate type with maximum precipitation occurring in July (95 mm) followed by August (89 mm). The weather remains pleasant in summer due to its location at high altitude. The hottest months are June and July with an average temperature of 24 °C and 23 °C respectively. Sometimes, the temperature rises to 29 °C. The coldest months are January and February with an average temperature of 5.3 °C and 6.6 °C respectively. Sometimes, the temperature falls to 1.1 °C, and at higher elevation, snowfall occurs (Fig. 2). The vegetation of the study area is subtropical humid and moist temperate type. The dominant tree species are *Pinus roxburghii* (Chir Pine) and *P. wallichiana* (Blue Pine). Due to the cool and humid condition, the vegetation is comprised of a wide variety of herbs, shrubs, and trees. The ground flora is composed of a number of angiosperms along with mosses and ferns.

The region embraced a diverse ethnic composition including Abbasi, Sudhans, Rajputs, and Gardazi. Among them, Abassi and Gardazi are the largest and well-settled tribes in the area. The whole population is Muslim. The majority of the population speaks the Hindko language, while Gojri and Urdu are also spoken. The major proportion of the indigenous community has very limited income sources. Majority of people are farmers, some people are job holders, some are labor, and few have their own business on a small scale. People also keep animals at their homes for livelihood. Few public health dispensaries are providing basic health facilities but people living at higher altitudes have limited access to them. They mainly depend on herbal remedies prepared at home or by traditional healers for primary health care.

### Sampling and plant identification

Several field trips were made in four different seasons (from August 2017–July 2018) following the method as reported previously [23]. Each medicinal plant species was collected in triplicates from different localities during guided tours. The specimens were properly dried, pressed, and mounted on standard herbarium sheets and voucher specimens were prepared following Jain and Rao’s methods [24]. Flora of Pakistan (https://www.eflora.com) [25, 26] was used for identification. For the correct family names, the APG IV (2016) [27] was followed, while for the accurate scientific name, ‘The Plant List (2013) [28] was used. The identified specimens were further confirmed in the AJ&K Medicinal and Aromatic Plant Herbarium PARC, Pakistan. The fully identified voucher specimens were then deposited in the herbarium of the Women University of Azad Jammu & Kashmir, Bagh.

### Data collection and analysis

Ethnomedicinal data were gathered from 74 informants including male (55%) and female (45%) using semi-structured interviews, questionnaire, group discussion, and field observation. The informants were selected on a random basis via convenience sampling and sample size was determined by Kadam and Bhalerao’s method [29]. For the preparation of the questionnaire Edward et al. method was used [30]. And ethical guidelines of the International Society of Ethnobiology (http://www.ethnobiology.net/) were strictly followed. In this regard, ethical approval was taken from the ethical committee of the Women University of Azad Jammu & Kashmir before starting surveys, while legal permission for conducting the survey was also taken from the representative of the municipality. Prior consent was taken from all the respondents following the participatory rural appraisal (PRA) approach as mentioned in the Kyoto Protocol after explaining the possible objective consequences of the study in the local language. Informants were not subjected to any clinical trial. Informants were classified into different categories like age, education level, and professions. The correctness of the ethnobotanical data was checked through triangulation. The data was then compared with the existing literature and analyzed both quantitatively and qualitatively.

### Ethnobotanical indices

For quantitative analysis various quantitative indices were applied including;

### Relative frequency citation

The frequency of citation (FC) was used to identify the most used plant species by the local inhabitants of the area. It was calculated by following Tardio and Pardo-de Santayana [5] and Vitalini et al. [31], using the following formula:

$$ \mathrm{RFC}=\frac{\mathrm{FC}}{\mathrm{N}} $$

where FC is respondents citing the use of specific species and *N* are the total respondents.

### Use value

The relative importance of particular plant species cited by all informants in a given area is quantitatively measured in terms of the use value. It was calculated by following Savikin et al. [32] using the following formula:

$$ \mathrm{UV}=\frac{\sum \mathrm{Ui}}{N} $$

where Ui is the number of citations or used reports by each respondent for a particular plant species and *N* is the total respondents.

### Informant consensus factor

The consensus between respondents and particular plant species used for each diseased category was tested by using informant consensus factor. It was figured out by following Vitalini et al., [5] using given formula:

$$ \mathrm{A}.\mathrm{ICF}=\frac{\mathrm{Nur}\hbox{-} \mathrm{Nt}}{\left(\mathrm{Nt}\hbox{-} 1\right)} $$

where ‘Nur’ represents the total number of used reports in each group of diseases, and ‘Nt’ represents the total species cited by all the informants for that group of ailments.

### Jaccard index

The similarity of indigenous knowledge among different communities was determined by using the Jaccard index (JI). It was calculated by following Gonzalez-Tejero et al. [33] using the given formula:

$$ \mathrm{JI}=\frac{\mathrm{C}\times 100}{\left(\mathrm{a}+\mathrm{b}\right)\hbox{-} \mathrm{c}} $$

where *a* is the species of the study area, *b* is the species recorded from the allied area, and *c* is the common species in both areas.

### Relative importance

Relative importance (RI) was figured out by following Khan et al. [34] using the given formula.

$$ {\displaystyle \begin{array}{c}\mathrm{RI}=\left(\mathit{\operatorname{Re}} lPH+\mathit{\operatorname{Re}} lBS\right)\times \frac{100}{2}\\ {}\operatorname{Re}1\mathrm{PH}=\frac{PH\; of\;a\; givn\kern0.17em plant}{Maximum\; PH\; of\; all\; reported\kern0.17em plant\kern0.17em species}\end{array}} $$

where PH is the pharmacological attribute of the selected plants and Rel PH is the relative number of pharmacological properties attributed to individual plant species.

$$ \operatorname{Re}1\mathrm{BS}=\frac{BS\; of\;a\; given\kern0.17em plant\;}{Maximum\; BS\; of\; all\; reported\kern0.17em plant\kern0.17em species} $$

BS is the number of body systems healed up by using single species and Rel BS is the relative number of body systems healed up by using a single species.

### Fidelity level

The fidelity level (FL) index was used to determine the most preferred species used to cure a particular disease as to treat the same ailment category with more than one plant species is also used. It was figured out after Friedman et al. [35], using the given formula:

$$ \mathrm{FL}=\frac{Np}{N}\times 100 $$

where *Np* is the number of respondents citing the use of species for a particular ailment and *N* is the total number of respondents citing the plants for any illness.

## Results and discussion

### Medicinal plants use and knowledge variation

The data on medicinal uses of plants was collected from 12 villages. Detail demographic data is given in Table 1. The females usually avoid participating and sharing knowledge with male interviewee due to communal restriction and Islamic instruction, which is also mentioned in other studies [36–38]. However, the women hold a wider competence regarding the traditional herbal recipes (5.36% species; 8.68% uses). A similar trend was also observed in other studies from Pakistan and abroad [39–41]. The older people (age ≤ 60) have more knowledge (6.46% species; 10.82% uses), followed by middle-aged people (age ≤ 40) (6.34% species; 9.50% uses) in comparison to adolescent informants (age ≤ 19) while it is inversely proportional to the level of education (Table 1). This might be the consequence of modernization and weak beliefs of young people regarding traditional remedies and due to changing lifestyles, development in modern medication, and urbanization [42, 43]. Similar findings are reported from other areas of Pakistan [44, 45] and elsewhere [46–48]. Illiterate native people are more accustomed to the usage of ethnomedicinal plants than literate people. The reason behind this is that educated people have very less interest in learning and practicing ethnobotanical knowledge. The same result was documented by other researchers in Pakistan [20, 49–51] and abroad [52, 53].

**Table 1:** Demographic information of the Informants

Variables	IC	Number	ANSRI	ANURI
Gender	Male	41	4.53	7.71
	Female	33	5.36	8.68
	Total	74		
Age-Class	19–40	17	4.17	3.46
	41–60	44	9.34	5.23
	Above 60	13	13.1	11.7
Education Level	Illiterate	12	6.59	4.23
	Elementary education	16	13.7	6.40
	Secondary education	18	13.1	6.02
	HSE	14	6.40	5.70
	Bachelor degree	9	17.1	4.92
	Higher education	5	11.5	6.91
Professions	THPs	12	21.5	10.4
	Midwives	07	12.4	7.36
	Herders	05	10.2	8.33
	Housewives	15	7.88	6.31
	Teachers	8	7.29	8.54
	Farmers	14	5.65	4.40
	Shopkeeper	04	4.18	3.98
	Students	06	4.31	3.04
	Labors	03	5.23	4.75

*IC* informants category, *ANSRI* average number of species reported by each informant, *ANURI* average number of use reported by each informant, *HSE* higher secondary education, *THPs* traditional health practitioners

### Local health care system

Throughout history, the role of traditional health practitioners (THPs) and midwives varies with time and culture, but even today, they are contributing significantly to the primary health care system, particularly among marginalized communities. THPs are usually aged males that use plants, animals, and minerals to treat various health disorders, whereas midwives are the elders and experienced females, which are familiar with pregnancy issues of women and treat them using diverse medicinal plants. Midwives are the integral component of a community that perform their important duties and provide essential support to women during delivery [54, 55]. Data given in Table 1 revealed that most of the information on ethnomedicinal uses of plant species of the study area were shared by (THPs), and midwives. The average number of species reported by THPs and midwives was 21.5 and 12.4, while they reported about 10.4% and 7.36% uses in respective order. Most of the traditional health practitioners were males who possess extensive information about therapeutic herbs and natural treatments which they use in herbal and other remedial preparations to cure diseases [56, 57]. However, as reported previously, traditional knowledge of plant resource utilization is declining due to changing lifestyle and more dependence on allopathic medicines [20, 51, 58, 59]. And similar trends were noted in the study areas.

### Diversity of ethnomedicinal flora

**Fig. 3:**
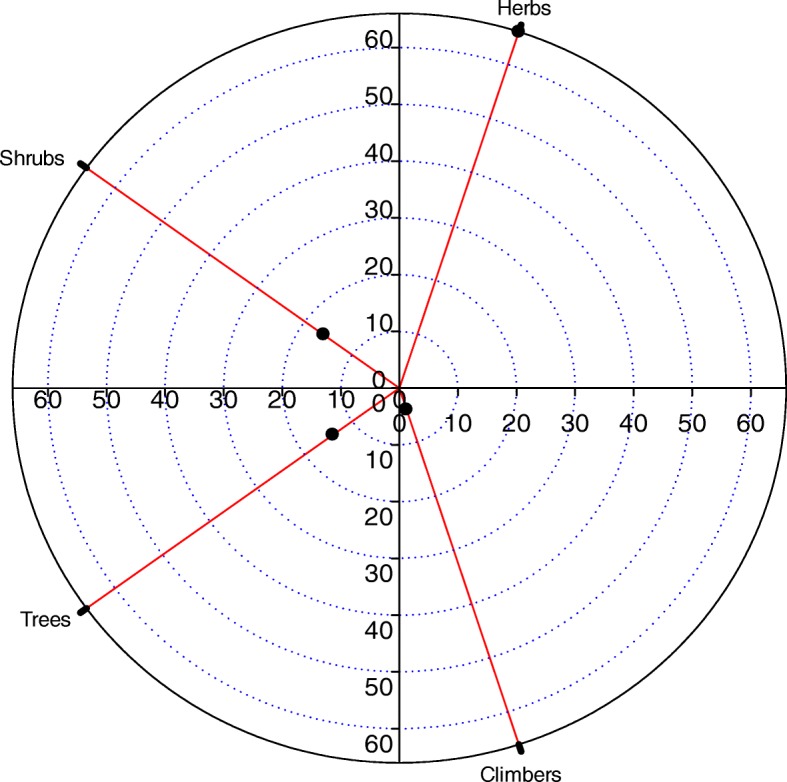
Life form distribution pattern of the reported plant species in the study area

**Fig. 4:**
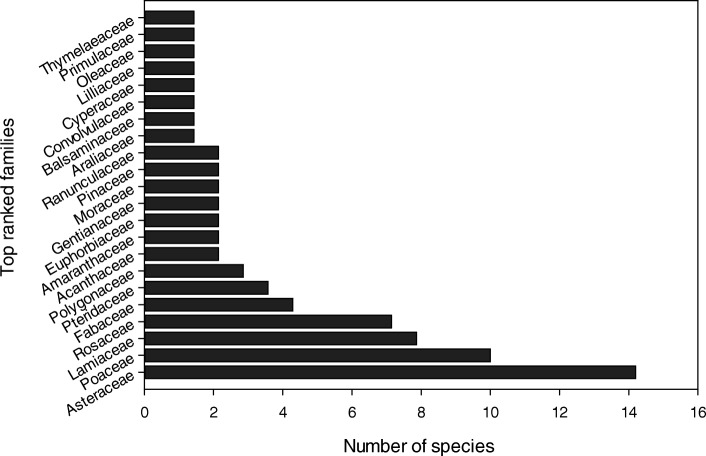
Top ranked families with number of species

A total of 140 species belonging to 55 families and 93 genera were reported (Table 2). Most of the documented ethnomedicinal plants species were herbs (66%) followed by shrubs (16%), trees (14%), and climbers (4%), (Fig. 3). This is because the study area is located in a dense forest zone at higher altitude where the herbs are abundantly distributed with few trees and shrubs. The bimodal rainfall and high availability of moisture might also be the reason. These findings are consistent with other studies [62–65, 69, 75, 76]. Among 22 families representing 2–20 plant species (Fig. 4), Asteraceae was the dominant family with 14.29% contribution of the total reported taxa, followed by Poaceae (10%), Lamiaceae (7.86%), Rosaceae (7.14%), Fabaceae (4.29%), and Pteridaceae (3.57%). All other families contributed less than 5% with percentages varying from 0.71–2.86%. The dominance of Asteraceae, Poaceae, Lamiaceae, and Rosaceae might be due to suitable habitat, favorable environmental conditions for the growth of the species belonging to these families, and more interactions of local communities with them in the study area. Therefore, traditional uses of plant species of these species are well recognized by the local inhabitants [6, 36, 66, 77, 78]. Additionally, majority of plant species belonging to the abovementioned families contain a variety of secondary metabolites and possess significant bioactivities, pharmacological, and organoleptic properties [79]. Floristic distribution of plant species in different families was analogues to previous reports from Pakistan and around the world [20, 36, 37, 74, 80–82].

**Table 2:** Medicinal uses of the reported taxa and their comparison with previous reports

Sr #	Family	Nomenclature		Habit	Medicinal uses				Previous reports
		Scientific name	Local name		Part used	Preparation	Application	Disease treated	
1	Acanthaceae	*Dicliptera roxburghiana Nees.*/*AF-110*	Churun	H	WP	PD	Internal	*Diabetes, *Tonic	1●, 2●, 3●, 4●, 5●, 6●, 7∎, 8●, 9●, 10●, 11●,12●, 13●, 14●, 15●, 16●,17●,18●, 19●, 20●, 21●,22●
					RT	EX	External	*Wounds	
		*Justicia vahlii Roth./AF-9*	Bhekkar	H	LF	IN	Internal	**Respiratory tract diseases**	1●, 2●, 3●, 4●, 5●, 6●,7●,8●, 9●, 10●, 11●, 12●,13●, 14●, 15●,16●,17●,18●, 19●, 20●, 21●,22●
		*Pteracanthus urticifolius (Wall. ex Kuntze) Bremek. /AF-48*	Blue Nettle	H	WP	EX	Internal	**Diuretic,** Stomach disorders, Ulcer	1●, 2●, 3●, 4●, 5●, 6●, 7●, 8●, 9●, 10●, 11●, 12●, 13●,14●, 15●, 16●, 17●,18●, 19●, 20●, 21●,22●
						DE	Internal	Sedative, Tonic	
2	Adoxaceae	*Viburnum grandiflorum* Wall. ex DC./AF-92	Guch	S	SD	JU	Internal	**Typhoid**	1●, 2●, 3◆, 4◆, 5●, 6∎, 7∎,8●,9●,10●, 11●, 12●, 13●, 14●, 15●,16●,17●, 18●, 19●, 20●, 21●, 22●
					FR	ET	Internal	*Stomachache	
3	Amaranthaceae	*Achyranthes aspera* L./AF-7	Puthkanda	H	LE	DE	External	*Toothache	1●, 2∎, 3∎, 4●, 5∎, 6●, 7●, 8●,9∎, 10●, 11●,12∎, 13●, 14∎, 15∎,16●,17●,18◆, 19∎, 20●, 21∎,22∎
					RT	EX	External	*Earache	
					WP	DE	Internal	*Pneumonia	
						EX	Internal	Dysentery	
		*Amaranthus viridis* L./AF-37	Ganyar	H	LE	VG	Internal	**Constipation**	1●, 2∎, 3●, 4●, 5∎, 6●, 7●, 8●, 9∎, 10●, 11●,12∎,13∎, 14●,15●, 16∎,17●, 18◆, 19∎, 20●, 21∎,22●
					ST	VG	Internal	Cough	
					SD	PD	Internal	Eye Vision	
		*Chenopodium ambrosioides* L. /AF-84	Bathu/Bathwa	H	WP	IN	Internal	*Measles, *Cough, Amenorrhea	1●, 2●, 3●, 4●, 5●, 6●, 7●, 8●, 9●, 10●, 11∎,12∎,13∎,14●,15●, 16●,17●, 18∎, 19●, 20●, 21∎,22●
					LE	PA	External	*Joint pain, *Backache	
						PD	Internal	*Cough, *Motion	
					SD	PD	Internal	*Diuretic, *Dropsy (oedema)	
4	Apocynaceae	*Nerium oleander* L*.* /AF-40	Kneer	S	LF	CH	External	Mouth disease	1●, 2∎, 3◆, 4◆, 5∎, 6●,7●,8●,9●, 10●, 11●, 1 2●,13●,14∎, 15●, 16●,17●, 18●, 19●, 20●, 21∎,22∎
					RT	PA	External	**Scorpion bite**	
					BA	EX	External	To kill wound worms	
5	Araliaceae	*Hedera nepalensis* K. Koch. /AF-135	Hurrbumbal/Betkal	E	LF	DE	Internal	**Diabetes**	1∎, 2●, 3◆, 4◆, 5◆,6●, 7●,8●,9●,10∎, 11●,12∎, 13●,14●, 15∎,16●, 17●, 18●, 19●, 20●, 21●,22●
						JU	Internal	*Indigestion, Ulcer	
		*Hydrocotyle* spp. L. /AF-114	Chamk wali boti	H	LF	EX	Internal	Fever, Bowel Complaints	
						EX	External	Cuts, **Burns**	1●, 2●, 3●, 4●, 5●, 6●, 7●, 8●, 9●, 10●, 11●,12●, 13●, 14●,15●,16●, 17●,18●, 19●, 20●, 21●, 22●
						PO	External	Syphilitic ulcers	
					WP	DE	Internal	Influenza, Hepatitis	
6	Aspleniaceae	*Asplenium dalhousiae* Hook. /AF-13	Niaroi	H	WP	JU	External	Blisters	1●, 2●, 3●, 4●, 5●, 6●,7●,6●, 8●, 9●, 10●, 11●, 12●, 13●, 14●, 15●, 16●, 17●, 18●, 19●, 20●, 21●,22●
							Internal	Cough	
					LE	EX	External	**Swelling,** Rickets	
7	Asteraceae	*Achillea millefolium* L. /AF-19	Sultani Booti / Kangi Booti	H	FL	EX	Internal	*Common Cold, *Flue, *Cough	1∎, 2●, 3∎, 4∎, 5●, 6●, 7●, 8∎,9●,10●, 11●, 12●, 13●, 14●, 15●, 16●, 17●, 18●, 19●, 20∎, 21●,22●
							External	*Arthritis	
					LE	PA	External	*Stop Bleeding, Wound Healing	
		*Artemisia vulgaris* L. /AF-55	Chaow	H	RT	EX	Internal	*Regulation of menstrual cycle	1∎, 2●, 3●, 4●, 5●, 6●, 7∎, 8●, 9●, 10∎, 11●, 12●, 13●, 14∎,15●,16●, 17●, 18●, 19●, 20●, 21●,22●
					WP	IN	Internal	*Cardiac problems	
		*Bidens biternata* (Lour.) Merr. & Sherff. /AF-79	Suryaly/Palouthi	H	LE	JU	Internal	**Sore infection**	1●, 2●, 3●, 4●, 5●, 6●, 7●, 8●, 9●, 10●, 11●,12●, 13●, 14●,15●,16●, 17●, 18●, 19●, 20●, 21●,22●
					RT	PA	External	Toothache	
		*Carpesium cernuum* L. /AF-43	Marchi	H	WP	EX	Internal	Cold, **Fever**	1●, 2●, 3●, 4●, 5●, 6●,7●, 8●, 9●, 10●, 11●, 12●, 13●, 14●, 15●,16●, 17●, 18●, 19●, 20●, 21●,22●
						JU	Internal	Sore throat	
					RT	EX	Internal	Antibacterial	
					SD	DE	Internal	Intestinal parasites, Abdominal pain	
		*Cichorium intybus* L. /AF-2	Kasni	H	RT	IN	Internal	Fever	1●, 2●, 3∎, 4●, 5●, 6●, 7∎, 8●, 9∎, 10●, 11∎, 12●, 13∎,14●,15●, 16∎,17●, 18●, 19●, 20●, 21●,22●
					LE	DE	Internal	Indigestion, *Typhoid, *Jaundice	
						PD	Internal	*Gout	
					LE	JU	Internal	*Gall Stones, *Gastrointestinal problems	
		*Cirsium vulgare* (Savi) Ten. /AF-127	Kandayara	H	WP	IN	External	**Joint disorders**	1●, 2●, 3●, 4●, 5●, 6●,7●, 8●, 9●, 10●, 11●, 12●, 13●,14●,15●, 16●,17●, 18●, 19●, 20●, 21●,21●,22●
						DE	Internal	Piles	
					RT	PO	External	Sore Jaws	
		*Conyza canadensis* (L.) Cronquist./AF-129	Kali Buti	H	WP	EX	Internal	Diuretic, *Cooling effect	1●, 2●, 3●, 4●, 5●, 6●,7∎,8∎,9●, 10●, 11●,12●,13●, 14∎,15●,16∎,17●,18●, 19●, 20●,21●,22●
						IN	Internal	*Sore throat, *Diarrhea, *nose bleeding	
					RT	DE	Internal	*Menstrual irregularities	
					LE	EX (Oil)	Internal	*Tonsils	
		*Galinsoga parviflora* Cav./AF-73	Peelibooti	H	WP	EX	External	*Skin disease, *Earache, *Scorpion bites	1●, 2●, 3●, 4●, 5●, 6●, 7●,8●,9●, 10●, 11●, 12∎, 13●,14●,15●,16●,17●,18●, 19●, 20●, 21●,22●
					LF	RB	External	*Skin inflammation	
						JU	External	*Blood clotting	
							Internal	*Dysentery	
		*Gerbera gossypina* (Royle) Beauverd./AF-27	Bhurjali/ Ladrun	H	LF	PA	External	**Wounds,** Skin Disease	1●, 2●, 3◆, 4∎, 5●, 6●,7●,8●,9●, 10●, 11●,12●,13●, 14●, 15●, 16●,17●,18●, 19●, 20●, 21●,22●
					AP	TE	Internal	*Nerve disorders	
		*Inula* spp. L./AF-95	Peeli Boti	H	WP	EX	Internal	Diabetes, Fever	1●, 2●, 3●, 4●, 5●, 6●, 7●,8∎,9●, 10●, 11●,12●, 13●, 14●, 15●,16●,17●,18●, 19●, 20●, 21●,22●
					RT	DE	Internal	**Digestive system disorders,** Asthma	
		*Matricaria matricarioides* (Less.) Porter ex Britton./AF-46	Pineapple-weed	H	WP	EX	Internal	**Vermifuge**	1●, 2●, 3●, 4●, 5●, 6●, 7●, 8●, 9●, 10●, 11●, 12●, 13●, 14●, 15●, 16●,17●,18●, 19●, 20●, 21●,22●
						TE	Internal	Cold, Fever	
					LF	IN	Internal	Stomach pain	
					SD	DE	Internal	Indigestion	
		*Myriactis wallichii* Less./AF-65	Safeed surajmukhi	H	LF	PA	External	**Wound healing**	1●, 2●, 3●, 4●, 5●, 6●,7●, 8●, 9●, 10●, 11●, 12●, 13●,14●, 15●, 16●,17●,18●, 19●, 20●, 21●,22●
		*Parthenium hysterophorus* L./AF-69	Gandibooti	H	LF	JU	Internal	*Fever, Constipation	1●, 2●, 3∎, 4∎, 5●, 6●,7●, 8●, 9●, 10●, 11●, 12●,13●,14●,15●, 16●,17●, 18◆, 19∎, 20●, 21∎,22●
						CH	External	Toothache	
					FL	PD	Internal	Diabetes	
					WP	DE	Internal	Dysentery, *Flue	
		*Phagnalon rupestre* DC./AF-51	Jijjo Booti	Sub-S	WP	DE	Internal	**Knee pain,** Renal stones	1●, 2●, 3●, 4●, 5●, 6●, 7●, 8●, 9●, 10●, 11●, 12●,13●, 14●, 15●,1 6●, 17●, 18●, 19●, 20●, 21●,22●
					FP	HB	Internal	Abdominal pain	
					LF	PD	External	Joints pain	
		*Prenanthes brunoniana* Wallex DC./AF-128	Himalayan Blue Sow-Thistle	H	WP	PO	External	**Wounds,** Sores	1●, 2●, 3●, 4●, 5●, 6●, 7●, 8●, 9●,10●, 11●,12●,13●, 14●, 15●, 16●,17●,18●, 19●, 20●, 21●, 22●
		*Sigesbeckia orientalis* L./AF-97	Yellow crown-head	C	LF	EX	External	Rheumatism, Paralysis	1●, 2●, 3●, 4●, 5●, 6●, 7●, 8●, 9●, 10●, 11●,12●, 13●, 14●, 15●,16●, 17●,18●, 19●, 20●, 21●,22●
						PA	External	**Wounds**	
					AP	DE	Internal	Hypertension	
						EX	External	Gout	
					WP	EX	External	Sore between toes	
		*Sonchus arvensis* L./AF-56	Dodhak/Dodhal	H	LF	PO	External	*Anti inflammation	1●, 2●, 3●, 4●, 5●, 6●, 7●, 8●, 9●, 10●, 11●, 12●, 13●,,14●, 15●, 16●,17●,18●, 19∎, 20●, 21●,22●
					WP	PA	External	*Wounds cleaning	
						JU	Internal	*Chronic fever	
					RT	DE	Internal	**Asthma**	
		*Sonchus oleracus* L./AF-106	Dodhak/Dodhal	H	LF	DE	Internal	*Constipation, *Body weakness	1●, 2●, 3●, 4●, 5∎, 6●,7●, 8●, 9●, 10●, 11●, 12●,13●,14●, 15●, 16●,17●,18●, 19●, 20●, 21●,22●
						PO	External	Swelling	
					WP	JU	Internal	*Ulcers	
						IN	Internal	Diarrhea	
					ST	LX	External	Warts	
		*Tagetes minuta* L./AF-139	Setbergha	H	FL	EX	Internal	*Fever	1●, 2●, 3●, 4●, 5∎, 6●,7∎, 8●, 9∎, 10●, 11●, 12∎, 13●,14●, 15●, 16●,17●,18●, 19●, 20●, 21●, 22●
					LF	JU	Internal	*Piles	
							External	*Earache, *Ophthalmic	
		*Taraxacum officinale* F.H. Wigg./AF-121	Hand	H	LF	VG	Internal	*Diabetes	1∎, 2●, 3∎, 4∎, 5∎, 6∎, 7●,8∎, 9∎,10●, 11●, 12●, 13●, 14●, 15●, 16∎,17●, 18●, 19●, 20∎, 21●, 22∎
						LX	Internal	*To stimulate Gallbladder, Indigestion	
					WP	JU	Internal	Liver disease, Jaundice	
					RH	DE	Internal	Jaundice	
8	Balsaminaceae	*Impatiens edgeworthii* Hook. f./AF-105	Tilchawli	H	WP	EX	Internal	*Urinary tract infection	1●, 2●, 3●, 4●, 5●, 6∎, 7●, 8●, 9●, 10●, 11●,12●,13●, 14●,15●, 16●,17●,18●, 19●, 20∎, 21●,22●
							External	*Burns	
							Internal	*Fever	
		*Impatiens glandulifera* Royle./AF-82	Tilcawli	H	RT	PA	External	*Cooling effect on hands and Foot	1∎, 2●, 3●, 4●, 5●, 6●,7●, 8●,9●, 10●, 11●,12●,13●,14●, 15●, 16●,17●,18●, 19●, 20◆,21●,22●
					LF	DE	Internal	Mental tension	
					FL	TE	External	*Eye wash	
9	Berberidaceae	*Berberis lycium* Royle./AF-4	Sumbal	S	LE	PA	External	*Bleeding, **Wound healing**	1∎, 2●, 3∎, 4∎, 5∎, 6∎, 7●,8∎, 9∎, 10∎, 11∎,12∎,13∎, 14∎,15∎, 16∎, 17●, 18●, 19●, 20●,21●,22∎
					RT	EX	Internal	*Joint Problems	
					BA	PD	Internal	Bleeding gums	
10	Boraginaceae	*Cynoglossum lanceolatum* Forssk./AF-23	Churuun	H	RT	EX	Internal	*Throat diseases	1∎, 2●, 3●, 4●, 5●, 6●, 7●,8∎, 9●, 10●, 11●,12●,13●, 14●,15●, 16●, 17●, 18●, 19●, 20∎, 21●,22●
					FR	CH	External	*Toothache	
					LE	PD	Internal	*Kidney disorder, *Tooth and gum diseases	
11	Brassicaceae	*Capsella bursa-pastoris* (L.) Medick./AF-94	Doddipatti	H	AP	VG	Internal	**Diarrhea**	1●, 2●, 3●, 4●, 5●, 6●,7●,8●, 9●, 10●, 11●,12●, 13●, 14●,15●, 16●, 17●,18●, 19∎, 20●, 21●,22●
					LE	DE	Internal	Menstrual disorders	
					WP	JU	Internal	* Nose bleeding	
12	Buxaceae	*Sarcococca saligna* (D. Don) Müll. Arg./AF-64	Niaroi/Ndroon	S	SH	EX	External	Joint pain	1∎, 2●, 3◆, 4◆, 5●, 6∎, 7∎, 8●, 9●, 10●, 11●, 12●, 13●, 14●, 15●, 16●, 17●, 18●, 19●, 20●, 21●, 22●
					RT	JU	Internal	**Gonorrhea**	
					LF	IN	Internal	Blood purification	
13	Campanulaceae	*Campanula pallida* Wall./AF-111	Beli Phool	H	WP	EX	Internal	**Dysentery,** Liver disorders	1●, 2●, 3●, 4●, 5●, 6●, 7●, 8●, 9●, 10●, 11●, 12●, 13●, 14●,15●,16●, 17●, 18●, 19●, 20●, 21●,22●
14	Cannabaceae	*Cannabis sativa* L./AF-83	Kamm/Bhang	H	LE	TE	Internal	*Joint problems	1●, 2∎, 3●, 4●, 5◆, 6●, 7●, 8∎, 9∎, 10●, 11●, 12∎, 13∎, 14∎, 15●, 16∎, 17∎,18∎, 19∎, 20●, 21∎,22●
					WP	DE	Internal	**Whooping cough**	
15	Convolvulaceae	*Convolvulus arvensis* L./AF-30	Speaker Booti	C	WP	VG	Internal	Skin Diseases	1●, 2∎, 3●, 4●, 5∎, 6●, 7∎, 8●, 9●, 10●, 11●, 12●, 13∎, 14●,15●, 16◆,17●,18◆, 19●,20●,21∎,22●
					RT	EX	External	**Dandruff**	
		*Ipomoea purpurea* (L.) Roth./AF-76	Eieer	C	SD	PD	Internal	Mental disorders, **Constipation,** Diuretic	1●, 2●, 3●, 4●, 5●, 6●,7●, 8●, 9●, 10●, 11●,12●, 13●, 14●,15●, 16●,17●,18●, 19●,20●, 21●,22●
					RT	EX	Internal	Syphilis	
					FL	EX	Internal	Laxative, Purgative	
16	Cyperaceae	*Cyperus serotinus* Rottb./AF-116	Deela Ghass	H	RT	EX	Internal	**Tonic,** Stimulant	1●, 2●, 3●, 4●, 5●, 6●, 7●,8●, 9●, 10●, 11●, 12●, 13●,14●,15●, 16●,17●,18●, 19●, 20●, 21●,22●
		*Eriophorum comosum* (Wall.) Nees. /AF-90	Berbaya	H	WP	PD	Internal	**Abdominal pain,** Kidney pain	1●, 2●, 3●, 4●, 5◆,6●, 7●,8●,9●, 10●, 11●,12●,13●, 14●, 15●, 16●,17●,18●, 19●, 20●, 21●,22●
17	Dryopteridaceae	*Dryopteris filix-mas* (L.) Schott. /AF-17	Kungi	H	FD	VG	Internal	**Diabetes**	1●, 2●, 3●, 4●, 5●, 6●, 7●, 8●, 9●, 10●, 11●,12●, 13●, 14●, 15●,16●, 17●, 18●, 19●, 20●, 21●,22●
					RT	EX	Internal	To treat Tapeworms	
							External	Muscle pain, Paralysis, Sciatica	
18	Ebenaceae	*Diospyros lotus* L. /AF-119	Amlook	T	FR	ET	Internal	*Stomach disease, *Fever	1●, 2●, 3●, 4●, 5●, 6●,7∎, 8 ∎,9●, 10∎, 11●, 12●,13∎, 14●, 15●, 16●,17●, 18●, 19●, 20●, 21●,22●
					TW	RB	External	*Toothache, *Gums and lips coloring	
19	Elaeagnaceae	*Elaeagnus umbellata* Thunb. /AF-77	Kankoli	S	SD	EX (Oil)	Internal	Breathing disorders, Lungs disease	1●, 2●, 3∎, 4∎, 5●, 6●,7◆,8●,9●,10●, 11●, 12●, 13●,14●, 15●, 16●,17●,18●, 19●, 20●, 21●,22●
					TW	RB	External	**Toothache**	
					FR	ET	Internal	*Mouth sore	
20	Euphorbiaceae	*Euphorbia indica* Lam. /AF-15	Dodhale/Dodhal	H	WP	DE	Internal	**Diarrhea,** Dysentery	1●, 2●, 3●, 4●, 5●, 6●, 7●, 8●, 9●, 10●, 11●, 12●,13●, 14●, 15●, 16●, 17●, 18●, 19●, 20●, 21●,22●
						LX	Internal	Purgative	
							External	Eye infection	
						PD	External	Oedema	
		*Euphorbia prostrata* Aiton. /AF- 49	Dodhal/Hazar Dani	H	WP	DE	Internal	Dysentery, **Diarrhea**	1●, 2●, 3●, 4●, 5●, 6●, 7●, 8●, 9●, 10●, 11●, 12●,13●, 14●, 15●, 16●,17●,18◆, 19●, 20●, 21◆,22●
					AP	IN	Internal	*Stomachache	
					LF	PD	External	*Headache	
		*Ricinus communis* L./AF-57	Hernoli	S	RT	EX	External	*Muscles weakness, *Gout disease	1∎, 2∎, 3●, 4●, 5∎, 6●, 7●, 8●, 9∎, 10∎, 11●,12●, 13●,14∎, 15∎, 16●, 18●, 19∎, 20●, 21●, 22●
					LF	EX	Internal	*To remove poisonous from body	
					SD	EX (oil)	Internal	*Scorpion bite	
							External	*Eye Disease, **Dandruff**	
21	Fabaceae	*Acacia nilotica (L.) Willd. ex Delile/AF-37*	Kikar	T	ST	Ash (PD)	External	*Eye Diseases	1●, 2∎, 3●, 4●, 5∎, 6●, 7●, 8∎, 9●, 10●, 11●, 12●, 13●, 14∎, 15●,16●, 17●, 18∎, 19∎, 20●, 21∎, 22●
					BA	DE	External	Toothache	
					FL	DE	External	*Earache	
					SD	PD	Internal	*Kidney pain, Diabetes	
							External	Toothpowder	
		*Desmodium elegans* DC./AF-31	Mangkit parang	S	WP	Extract	Internal	*Diarrhea	1●, 2●, 3●, 4●, 5●, 6●, 7●, 8●, 9●, 10●, 11●,12●,13●, 14∎, 15●, 16●,17●,18●, 19●, 20●, 21●,22●
					LE	Extract	External	*Eye Infection	
							Internal	*Cough, *Fever, *Vomiting	
					RT	Powder	External	*Scorpion and Snake bites	
		*Indigofera heterantha* Wall.ex Brandis./AF-33	Jand	S	BR	PD	Internal	**Whooping cough**	1●, 2●, 3●, 4●, 5●, 6◆,7●, 8∎,9●, 10∎, 11●,12∎, 13●, 14∎, 15●, 16●,17●,18●, 19●, 20∎, 21●,22●
					LF	EX	Internal	Mouth infection	
		*Lespedeza juncea* (L. f.) Pers./AF-133	Silky bush-clover	H	SH	DE	Internal	Dysentery, Diarrhea	1●, 2●, 3◆, 4◆,5●, 6●,7●,8●, 9●, 10●, 11●, 12●, 13●,14●,15●, 16●,17●,18●, 19●, 20●, 21●,22●
					WP	DE	External	*Skin ulcers, *Toothache	
		*Medicago lupulina* L./AF-132	Sirri	H	SD	PD	Internal	**Indigestion**	1●, 2●, 3●, 4●, 5●, 6●, 7●, 8●,9●,10●, 11●,12●,13●,14●,15●, 16●,17●, 18●, 19●, 20●, 21●,22●
					WP	EX	Internal	Antibacterial	
		*Trifolium pratense* L./AF-42	Trapetra	H	FL and LF	EX	Internal	Minimize menopause symptoms	1●, 2●, 3●, 4●, 5●, 6●, 7●, 8●, 9●, 10●, 11●, 12●, 13●,14●,15●, 16∎,17●,18●, 19●, 20●, 21●,22●
					WP	PO	External	*Breast cancer	
						DE	Internal	*Cancer, *Whooping Cough, *Gout disease	
22	Fagaceae	*Quercus incana* W. Bartram./AF-32	Rein	T	SD	PD	Internal	*Diuretic	1∎, 2●, 3∎, 4∎, 5●, 6●, 7∎,8◆,9●, 10●, 11∎, 12●, 13●,14●, 15∎, 16●, 17●,18●, 19●, 20●, 21●, 22●
					GL	DE	Internal	Joint swelling, Dysentery	
					STb	PD	External	*Skin ulcer	
						DE	Internal	Throat pain	
23	Gentianaceae	*Gentianodes olivieri* (Griseb.) Omer, Ali & Qaiser./AF-44	Neeli Booti	H	WP	DE	Internal	Jaundice, Cough	1●, 2●, 3●, 4●, 5●, 6●,7●, 8●, 9●, 10●, 11●,12●,13●, 14●, 15●,16●,17●, 18●, 19●, 20●, 21●,22●
						PD	Internal	**Throat problem**	
		*Swertia cordata* (Wall. ex G. Don) C.B. Clarke./AF-26	Plamas	H	WP	EX	Internal	Pneumonia fever, Throat problems, **Malarial fever**	1●, 2●, 3●, 4●, 5●, 6●,7●, 8●, 9●, 10●, 11●, 12●, 13●, 14●,15●, 16●, 17●,18●, 19●, 20∎, 21●, 22●
						IN	Internal	To kill intestinal worms	
						PD	Internal	Tonic	
		*Swertia paniculata* Wall./AF-50	Plamas/Jabba jarri	H	WP	EX	Internal	*Malarial Fever, *Diarrhea	1●, 2●, 3●, 4●, 5●, 6∎, 7●, 8●, 9●, 10●, 11●, 12●,13●, 14●, 15●, 16●, 17●,18●, 19●, 20●, 21●, 22●
						DE	Internal	*Tonic	
24	Hypericaceae	*Hypericum perforatum* L./AF-59	Sharan Gulab	H	SH	DE	Internal	*Anxiety	1●, 2●, 3∎, 4∎, 5●, 6●,7●, 8●, 9●, 10●, 11●, 12●,13●, 14●, 15●, 16●,17●, 18●, 19●, 20●, 21●,22●
					WP	EX	Internal	*Depression	
							External	*Bruises, Wounds, *Intestinal problems	
					FL	IN	External	Swelling, *Sunburns	
25	Lamiaceae	*Ajuga bracteosa* Wall. ex Benth./AF-20	Thandi Jarri/Ratti Booti	H	LE	DE	Internal	Skin Infection, **Stomach problem**	1∎, 2∎, 3∎, 4∎, 5●, 6●,7●,8◆, 9●, 10∎, 11●,12●, 13●, 14●, 15∎, 16●, 17∎, 18●, 19●, 20●,21●,22●
					WP	EX	Internal	Jaundice, *Ulcer	
		*Ajuga parviflora* Benth./AF-21	Thandi Jarri	H	LE	EX	Internal	**Gastric problem**	1●, 2●, 3●, 4●, 5●, 6●,7●,8●,9●, 10●, 11●, 12●, 13●, 14●, 15●, 16●, 17●, 18●, 19●, 20●, 21●,22●
					WP	EX	Internal	Hypertension, Headache	
		*Isodon rugosus* (Wall. ex Benth.) Codd./AF-80	Chitta Manja	S	SD	DE	Internal	Blood purifier	1●, 2●, 3∎, 4∎, 5●, 6●, 7●, 8●, 9●, 10●, 11●, 12●, 13●, 14∎, 15◆,16●,17●,18●, 19●,20●, 21●, 22●
					SH	EX	Internal	Abdominal pain	
					LF	PD	Internal	*Digestive problem	
						PA	External	Blood clotting	
		*Mentha arvensis* L./AF-28	Podina	H	LF	DE	Internal	**Stomach acidity,** Indigestion, Vomiting	1●, 2∎, 3●, 4●, 5●, 6●,7◆,8◆,9∎,10●, 11●,12●, 13●, 14●, 15●, 16●,17∎, 18●, 19●, 20●, 21●,22●
						EX	Internal	Dysentery, Diarrhea	
		*Mentha longifolia* (L.) Huds./AF-29	Bareena	H	LF	DE	Internal	Digestive disorders, Abdominal disorders	1∎, 2◆, 3●, 4●, 5∎, 6●,7∎,8●,9∎,10◆, 11∎, 12●, 13∎, 14◆, 15∎, 16◆, 17∎, 18●, 19●, 20●, 21●,22●
						PD	Internal	Gastrointestinal problems	
						TE	Internal	*Headache	
		*Micromeria biflora* (Buch.-Ham. ex D. Don) Benth. /AF-93	Chai booti	H	LF	JU	Internal	**Digestive disorders**	1●, 2●, 3●, 4●, 5∎, 6●, 7●, 8●, 9●, 10●, 11●,12∎, 13●, 14●,15∎, 16●,17∎, 18●, 19●, 20●, 21●,22●
					RT	PA	External	*Toothache	
					WP	JU	Internal	*Sinus infection	
		*Nepeta laevigata* (D. Don) Hand.-Mazz. /AF-125	Jangli Bhaker	H	WP	PD	Internal	*Fever, *Headache	1●, 2●, 3∎, 4∎, 5●, 6●,7●, 8●, 9●, 10●, 11●,12●,13●,14●, 15●, 16●,17●, 18●, 19●, 20●, 21●,22●
					SD	IN	Internal	*Dysentery	
		*Origanum vulgare* L./AF-62	Ban ajwain	H	WP	JU	Internal	Stomachache	1●, 2●, 3●, 4●, 5●, 6●, 7●, 8●, 9●, 10●, 11●, 12∎, 13●,14●,15∎, 16●,17●,18●, 19●, 20●, 21●,22●
						DE	External	**Skin Infection**	
						EX (oil)	External	*Pain reliever	
					SH	CH	External	Toothache	
		*Plectranthus rugosus* Wall.ex Benth. /AF-34	Peemar	S	LF	CH	External	*Toothache	1∎, 2●, 3●, 4●, 5●, 6●, 7●, 8∎, 9∎, 10●, 11●, 12●, 13●, 14●, 15●, 16●,17●, 18●, 19●, 20●, 21●,22●
					RT	DE	Internal	*Liver tonic	
		*Prunella vulgaris* L./AF-72	Kathri	H	LF	DE	Internal	*Sore throat	1∎, 2●, 3∎, 4∎, 5●, 6∎, 7●,8●, 9●, 10●, 11●,12●,13●, 14●, 15●, 16●, 17●, 18●, 19●, 20∎, 21●,22●
						PA	External	Skin infection	
					WP	PD	External	*Joint pains	
						DE	Internal	Heart disease	
		*Salvia lanata* Roxb./AF-126	Kathra	H	IN	VG	Internal	*Cough	1●, 2●, 3∎, 4∎, 5●, 6●,7●,8●,9●, 10●, 11●,12●,13●,14●,15●, 16●,17●, 18●, 19●, 20●, 21●, 22●
					LF	PO	External	Wounds, Itching	
					WP	EX	Internal	*Abdominal worms, *Motion	
26	Lauraceae	*Machilus odoratissimus* Nees./AF-104	Chaan	T	AP	EX	Internal	**Diabetes,** Epilepsy, Cardiovascular diseases	1●, 2●, 3●, 4●, 5●, 6●, 7●,8●, 9●, 10●, 11●, 12●, 13●, 14●, 15●, 16●, 17●,18●, 19●, 20●, 21●,22●
27	Lilliaceae	*Allium cepa* L./AF-137	Piyaz	H	BL	JU	Internal	*Diarrhea	1●, 2∎, 3●, 4●, 5∎, 6●,7●, 8●,9∎, 10●, 11∎, 12●, 13∎, 7●, 15●, 16∎, 17●, 18●, 19●, 20●, 21,22●
						DE	External	*Dandruff, *Hair fall	
						HR	External	*To remove water from wounds	
		*Allium sativum* L. /AF-134	Thoom	H	BL	PA	External	*Hair growth	1●, 2∎, 3●, 4●, 5∎, 6●, 7●, 8●, 9∎, 10●, 11●,12●, 13●, 14∎, 15●, 16●, 17●, 18●, 19●, 20●,21●,22●
						DE	Internal	*Common cold	
						CH	External	**Hypertension**	
						EX	External	*Joint pain	
					LE	PD	Internal	*Stomach problems	
28	Lythraceae	*Punica granatum* L*.*/AF-66	Darun/ Jangle annar	S	SD	JU	Internal	*Diabetes	1●, 2∎, 3∎, 4∎, 5●, 6●,7∎, 8∎, 9∎,10∎, 11∎, 12∎,13∎, 14∎,15●, 16∎,17●, 18●, 19●, 20●, 21●, 22∎
					LF	PA	External	Tooth pain	
					FR	ET	Internal	Jaundice	
					BR	DE	Internal	Antithelmintic	
29	Malvaceae	*Malva parviflora* L./AF-74	Sonchal	H	LF	VG	Internal	**Constipation**	1●, 2●, 3●, 4●, 5∎, 6●, 7●, 8●, 9●, 10●, 11●,12◆13●,14●, 15●, 16∎,17●, 18◆, 19●, 20●, 21◆,22●
						DE	Internal	Cough	
					WP	PO	External	*To remove swelling	
					RT	DE	External	*Dandruff	
30	Meliaceae	*Melia azedarach* L./AF-6	Daraik	T	FR	EX	Internal	Diabetes, Blood purification	1●, 2∎, 3●, 4●, 5∎, 6●, 7●, 8●, 9◆, 10∎, 11●,12◆,13∎,14∎,15∎,16●, 17∎, 18◆, 19∎, 20●, 21∎,22●
					LB	EX	Internal	**Blood purification**	
					LF	EX	External	*Tonic, Antiseptic, Hair Fall	
31	Moraceae	*Ficus carica* L./AF-25	Phagwara	S	FR	EX	Internal	Mouth ulcers, Inflammation	1●, 2∎, 3∎, 4∎, 5∎, 6●, 7●, 8●, 9●, 10●, 11∎,12∎,13∎, 14∎, 15●,16∎, 17●, 18●, 19●, 20●, 21●,22◆
					LF	LX	External	Insect bites, **Warts**	
						DE	Internal	Piles	
					FR	ET	Internal	Constipation	
		*Ficus palmata* Forssk./AF-10	Phagwara/ Injeer	Tree	FR	ET	Internal	*Stomach disorders, Constipation	1●, 2∎, 3∎, 4∎, 5●, 6●, 7∎,8●,9∎, 10∎, 11∎, 12●, 13●,14●, 15●,16●,17∎, 18●, 19●, 20●, 21●,22●
					LF	LX	External	Skin infection, *Epilepsy	
		*Morus alba* L./AF-115	Shehoot	T	FR	EX	Internal	*Sexual disorders	1●, 2∎, 3●, 4●, 5∎, 6●,7●, 8∎, 9●, 10∎, 11∎, 12●, 13∎, 14●,15●,16∎,17●, 18∎, 19∎, 20●, 21∎,22●
						JU	Internal	*Body weakness, Chest Infection	
32	Oleaceae	*Jasminum grandiflorum* L. /AF-36	Jasmine/Chambeli	S	FL	EX	Internal	**Breast cancer**	1●, 2●, 3●, 4●, 5●, 6●, 7●, 8●, 9●,10●, 11●,12●,13●, 14●, 15●, 16●,17●,18●, 19●, 20●, 21●, 22●
						JU	External	Eye disorders	
						IN	Internal	Fever	
					LF	CH	External	Mouth ulcer, Dental pain	
					RT	EX (oil)	External	Headache	
						PA	External	Scabies	
		*Olea ferruginea* Royle./AF-8	Kaow	T	LF	CH	External	**Mouth infection**	1∎, 2∎, 3●, 4●, 5∎, 6●,7●,8∎, 9∎, 10∎, 11●,1 2●,13∎, 14◆,15∎,16◆,17●, 18●, 19●, 20●, 21●,22●
						TE	Internal	Digestive disorders, Diabetes	
					FR	EX	External	*Hair growth	
33	Onagraceae	*Oenothera rosea* L’Hér.ex Aiton. /AF-58	Buti/ Seh Davi	H	LF	IN	Internal	Kidney disorders	1●, 2●, 3◆, 4◆, 5●, 6●, 7∎, 8●,9●,10●, 11●, 1 2●,13●, 14●, 15●, 16●, 17●, 18●, 19●, 20●, 21●,22●
					RT	PD	Internal	*Body weakness	
34	Oxalidaceae	*Oxalis corniculata L.*/AF-41	Khati Buti	H	WP	ET	Internal	**Jaundice**	1●, 2∎, 3●, 4●, 5●, 6●,7∎, 8●,9∎,10∎, 11●,12∎,13∎, 14●,15∎,16∎,17∎, 18◆, 19∎, 20∎, 21∎, 22∎
					LF	CH	External	Toothache	
						DE	Internal	Diarrhea	
						ET	Internal	Blood purification	
35	Pinaceae	*Cedrus deodara* (Roxb. ex D. Don) G. Don./AF- 61	Dayar	T	ST	EX (oil)	External	**Skin disorders (eczema),** *Joint pain	1●, 2●, 3●, 4●, 5●, 6∎, 7●, 8●, 9●, 10●, 11∎, 12●, 13●, 14∎, 15 ●,16●,17●, 18●, 19●, 20●, 21●,22●
							Internal	*Digestive disorders	
					ND	PA	External	*Swelling, *To clean wounds, Chest infection	
		*Pinus roxburghii* Sarg./AF-87	Chir	T	LF	DE	Internal	*Flue	1●, 2●, 3∎, 4∎, 5∎, 6●, 7∎, 8●, 9●,10∎, 11●, 12∎, 13●, 14●, 15●, 16●,17∎,18●, 19●, 20●, 21●,22●
					RS	PO	External	**Wound healing,** *Cracked Heels	
							Internal	*Joint diseases, Digestive disorders, *Scorpion Bite	
					WP	Oil	Internal	*Nose bleeding, *Flue	
		*Pinus wallichina* A.B. Jacks./AF-16	Biyar	T	RS	PO	Internal	*Cough	1●, 2●, 3∎, 4∎, 5●, 6●,7●,8●,9●, 10●, 11∎, 12●,13∎, 14●, 15●,16●,17●,18●, 19●, 20∎, 21●,22●
							External	Wound healing	
						IN	Internal	*Expulsion of worms	
						EX	Internal	*Diuretic, *Kidney problem	
36	Plantaginaceae	*Plantago lanceolata* L./AF-86	Chamchi ptra/ Ispagol	H	FL	IN	Internal	Dysentery	1●, 2∎, 3∎, 4∎, 5◆,6●, 7∎, 8●, 9●, 10●, 11∎, 12●, 13∎, 6●, 14●, 15◆, 16∎, 17●, 18●, 20◆, 21●, 22●
					SD	PD	Internal	**Diarrhea**	
					LF	PA	External	Cuts, *Inflammation	
37	Platanaceae	*Planatus orientalis* L./AF-123	Chinar	T	BA	JU	Internal	*Snake and *Scorpion bite	1●, 2●, 3●, 4●, 5●, 6●, 7●, 8●, 9●, 10●, 11●, 12●,13●, 14∎, 15●, 16●,17●,18●, 19●, 20● 21●, 22●
						DE	Internal	*Dysentery	
					LF	PA	External	*Wound healing	
						DE	Internal	*Dysentery	
						PD	Internal	*Teeth pain	
38	Poaceae	*Arthraxon prionodes* (Steud.) Dandy/AF-100	Kah	H	WP	DE	Internal	**Liver disease,** Nervous system regulator	1●, 2●, 3●, 4●, 5●, 6●, 7●, 8●, 9●, 10●, 11●, 12●, 13●, 14●,15●, 16●, 17●, 18●, 19●, 20●, 21●,22●
		*Aristida cyanantha* Nees ex Steud./AF-122	Common Ghass	H	WP	Ash (PD)	External	Burns, **Skin infection**	1●, 2●, 3●, 4●, 5●, 6●, 7●, 8●, 9●, 10●, 11●, 12●, 13●, 14●,15●, 16●, 17●, 18●, 19●, 20●, 21●,22●
					LE	EX	Internal	Antithelmintic	
		*Bromus catharticus* Vahl./AF-68	Jarun ghass	H	RT	EX	Internal	Purgative	1●, 2●, 3●, 4●, 5●, 6●, 7●, 8●, 9●, 10●, 11●, 12●, 13●, 14●,15●, 16●, 17●, 18●, 19●, 20●, 21●,22●
					WP	EX	External	**Skin disorders**	
		*Chrysopogon gryllus* (L.) Trin./AF-89	BunchGrass	H	LE	DE	Internal	**Fish Poisonings**	1●, 2●, 3●, 4●, 5●, 6●, 7●, 8●, 9●, 10●, 11●, 12●, 13●, 14●,15●, 16●, 17●, 18●, 19●, 20●, 21●,22●
		*Cymbopogon martini* (Roxb.) Will. Watson./AF-140	Munyara Ghass	H	WP	DE	Internal	**Diarrhea,** Intestinal worms	1●, 2●, 3●, 4●, 5●, 6●, 7●, 8●, 9●, 10●, 11●, 12●, 13●, 14●,15●, 16●, 17●, 18●, 19●, 20●, 21●,22●
						IN	Internal	Anorexia	
					LE	PA	External	Skin diseases	
					ST	PA	External	Scabies	
		*Cynodon dactylon* (L.) Pers./AF-18	Khabal	H	IN	PA	External	*Skin infection	1●, 2∎, 3●, 4●, 5∎, 6∎, 7∎, 8●,9∎, 10∎, 11●,12●, 13●, 14●, 15●, 16∎, 17●,18∎, 19∎, 20●, 21∎, 22●
					WP	JU	Internal	*Menstrual prolonged duration, Stomach acidity	
							External	Eye Infection	
						PA	External	*Wounds healing	
		*Dactylis glomerata* L./AF-107	Billi Ghass	H	LE	EX	Internal	**Kidney problem,** Bladder ailment	1●, 2●, 3●, 4●, 5●, 6●, 7●, 8●, 9●, 10●, 11●, 12●, 13●, 14●,15●, 16●, 17●, 18●, 19●, 20●, 21●,22●
					WP	EX	Internal	Rickets	
					PL	EX	Internal	Premenstrual syndrome	
		*Dichanthium annulatum* (Forssk.) Stapf./AF-118	Golgen beared Ghass	H	WP	EX	Internal	*Dysentery, *Menorrhagia	1●, 2●, 3●, 4●, 5●, 6●,7●, 8●, 9●, 10●, 11●, 12●, 13●, 14●, 15●,16●,17●, 18∎, 19●, 20●, 21∎,22●
					RT	EX	Internal	*Blood purification	
		*Eleusine indica* (L.) Gaertn./AF-131	Madhani ghass	H	WP	PA	External	*Stop bleeding	1●, 2●, 3●, 4●, 5●, 6●, 7●, 8●, 9●, 10●, 11●, 12●, 13●,14●,15●, 16●,17●, 18∎, 19∎, 20●, 21∎,22●
					LF	JU	Internal	*Anthelmintic	
					RT	DE	Internal	*Asthma	
		*Oplismenus compositus* (L.) P. Beauv./AF-130	Running mountaingrass	H	AP	EX	External	**Snake bite**	1●, 2●, 3●, 4●, 5●, 6●, 7●, 8●, 9●, 10●, 11●, 12●, 13●, 14●,15●, 16●, 17●, 18●, 19●, 20●, 21●,22●
		*Pennisetum orientale* Rich. /AF-35	Siliak ghass/Haati Gaas	H	AP	EX	External	*Snake bite	1●, 2●, 3●, 4●, 5●, 6●, 7●, 8●, 9●, 10●, 11●, 12●, 13●, 14●,15●, 16●, 17●, 18●, 19●, 20●, 21●,22●
		*Saccharum spontaneum* L. /AF-101	Kai	H	WP	JU	Internal	*Cough, *Abdominal pain	1●, 2●, 3●, 4●, 5●, 6●,7∎, 8●, 9●, 10●, 11●, 12●, 13●, 14●, 15●, 16●,17●, 18●, 19●, 20●, 21∎, 22●
					RT	EX	Internal	*Piles, *Sexual weakness, *Dyspepsia	
						DE	Internal	*Kidney stones	
		*Setaria viridis* (L.) P. Beauv./AF-113	Kera Ghass	H	SD	PD	Internal	*To remove extra fats from body	1●, 2●, 3●, 4●, 5●, 6●, 7∎, 8●, 9●, 10●, 11●,12●, 13●, 14●, 15●, 16●,17●, 18●, 19●, 20●, 21●,22●
						DE	Internal	*Diuretic	
					WP	IN	External	*Bruises	
		*Sorghum halepense* (L.) Pers./AF-102	Barun ghass	H	RT	EX	Internal	Indigestion	1●, 2●, 3●, 4●, 5∎, 6●,7∎, 8●, 9●, 10●, 11●,12●,13●, 14●, 15●, 16●,17●,18∎, 19●, 20●, 21●, 22●
					SD	PD	Internal	*Diuretic	
					LF	PA	External	*Blood clotting, *Antiseptic	
					AP	EX	Internal	*Abortion	
39	Polygonaceae	*Persicaria capitata* (Buch.-Ham. ex D. Don) H. Gross./AF-125	Pink bubble	H	AP	DE	Internal	Fever, Diarrhea	1●, 2●, 3●, 4●, 5●, 6●, 7●, 8●, 9●,10●, 11●,12◆,13●,14●,15●,16●,17●,18●, 19●, 20∎, 21●,22●
							External	*Eye diseases	
					WP	EX	Internal	*Diuretic, *Hypothermia	
					RT	EX	Internal	Urinary tract infection	
		*Polygonum hydropiper* L./AF-38	Knotweed/Marsh weed	H	WP	DE	Internal	Menorrhagia	1●, 2●, 3●, 4●, 5●, 6●, 7●, 8●, 9●, 10●, 11●,12●, 13●,14●,15●,16●,17●, 18●, 19●, 20●, 21●, 22●
						EX	Internal	**Joints pain,** Neurodegenerative disorders	
					LF	JU	Internal	Liver pain	
					SD	PD	Internal	Laxative	
					RT	EX	Internal	Tonic	
		*Rumex dentatus* L. /AF-88	Hullah/ Jangli palak	H	LF	PA	External	Antiseptic	1●, 2●, 3∎, 4◆, 5∎, 6●,7●,8●, 9●, 10●, 11●, 12●,13●,14●,15●, 16●, 17∎, 18∎, 19●, 20●, 21●, 22●
						PD	External	Wound Healing	
						RB	External	*Itching caused by ***Utrica dioica***	
		*Rumex hastatus* D. Don./AF-63	Chukri/Harfali	S	AP	RB	External	Scabies	1●, 2●, 3∎, 4∎, 5●, 6◆,7∎, 8●,9∎,10●, 11●, 12∎, 13∎, 14∎, 15∎, 16●,17∎,18●, 19●, 20●, 21∎, 22●
					LF	EX	Internal	*Jaundice	
40	Primulaceae	*Androsace rotundifolia* Hardw./AF-14	Thandi jari	H	LE	EX	Internal	**Stomach diseases,** Menstrual problem	1●, 2●, 3◆, 4◆, 5●, 6●, 7●, 8●, 9●, 10●, 11●, 12●, 13●, 14●,15●, 16●,17●, 18●, 19●, 20◆,21●,22●
					RH	EX	External	Eye disease	
		*Myrsine africana* L. /AF-22	Gogel	S	LF	IN	Internal	*Stomachache	1●, 2●, 3∎, 4∎, 5●, 6●,7●, 8●, 9●, 10●, 11●,12◆,13●,14◆,15●,16●,17●, 18●, 19●, 20●, 21●,22●
						DE	Internal	Blood Purifier	
					FR	ET	Internal	**To remove intestinal Tapeworms,** *Mouth Infection	
						PD	Internal	*Stomachache	
41	Pteridaceae	*Adiantum caudatum* L./AF-124	Maneria	H	FD	EX	External	**Wound healing,** Skin diseases	1●, 2●, 3●, 4●, 5●, 6●, 7●, 8●, 9∎, 10●, 11●, 12●, 13●, 14●, 15●, 16●, 17●, 18●, 19●,20●, 21●,22●
						JU	Internal	Cough, Diabetes, Migraine	
		*Adiantum tenerum* Sw./AF-11	Hansraj	H	FD	PO	External	**Snake bite**	1●, 2●, 3●, 4●, 5●, 6●,7●,8●,9∎, 10●, 11●, 12●,13●, 14●, 15●, 16●, 17●, 18●, 19●,20●, 21●,22●
						DE	Internal	Fever, To kill intestinal worms	
					WP	EX	Internal	Cough, Fever, Pneumonia	
		*Onychium japonicum* (Thunb.) Kunze./AF-108	Carrot Fern	H	WP	EX	Internal	Common cold, Dysentery, **Jaundice**	1●, 2●, 3●, 4●, 5●, 6●, 7●, 8●, 9●, 10●, 11●,1 2●,13●, 14●, 15●,16●,17●,18●, 19●, 20●, 21●,22●
					LF	JU	External	Hair fall	
		*Pteris cretica* L. /AF-60	Cretan brake	H	FD	PA	External	Wound healing	1●, 2●, 3◆, 4◆, 5●, 6●,7◆,8●,9●,10●, 11●, 12●,13●, 14●,15●, 16●,17●,18●, 19●, 20●, 21●,22●
					WP	DE	Internal	*Cough	
		*Pteris vittata* L./AF-45	Nanore	H	WP	PA	External	*Bone Fracture	1●, 2●, 3●, 4●, 5∎, 6●,7 ●, 8●, 9●, 10●, 11●, 12●, 13●,14●,15●,16●,17●, 18●, 19●, 20●, 21●,22●
						EX	Internal	*Hypotonic	
					FD	PA	External	*Antibacterial, *Antifungal	
42	Ranunculaceae	*Clematis grata* Wall./AF-78	Bailari	C	RT	EX	Internal	*Bile disorders	1●, 2●, 3●, 4●, 5●, 6●, 7●, 8●, 9●, 10●, 11●, 12∎, 13●, 14●,15●, 16●, 17●,18●, 19●, 20●, 21●,22●
					LE	TE	Internal	*Scanty lacto genesis	
		*Ranunculus arvensis* L./AF-112	Jungli dhaniya	H	WP	EX	Internal	Asthma, **Arthritis,** Hay fever	1●, 2●, 3●, 4●, 5●, 6●, 7●,8●,9∎, 10●, 11●,12●,13●,14●, 15●, 16●, 17●,18●, 19●, 20●, 21●, 22●
						DE	Internal	To Kill Intestinal Worms	
						LF	EX	External	
		*Ranunculus muricatus* L./AF-120	Kor kandoli	H	AP	CK	Internal	**Asthma**	1●, 2●, 3∎, 4∎, 5●, 6●,7●, 8●,9●, 10●, 11●, 12●,13●,14●, 15●, 16∎,17●,18●, 19∎, 20●, 21●, 22●
					WP	EX	Internal	*Gout, Fever	
43	Rosaceae	*Duchesnea indica* (Andrews) Teschem./AF-39	Budimeva/ Surkh Akhra	H	FR	ET	Internal	*Kidney stone	1●, 2●, 3●, 4∎, 5●, 6●, 7●, 8●, 9●, 10●, 11●,12∎,13●, 14∎,15◆,16∎,17●,18●, 19●, 20●, 21●,22●
					LF	DE	Internal	Sexual weakness, Mental disorders	
		*Fragaria nubicola* (Hook. f.) Lindl.ex Lacaita./AF-136	Budi meva	H	RT	PD	Internal	Urinary disorder	1●, 2●, 3∎, 4∎, 5●, 6∎, 7●, 8●, 9●,10●, 11∎, 12●, 13∎,14●, 15∎, 16●,17●, 18●, 19●, 20∎, 21●,22●
					FR	JU	Internal	*Diabetes, *Sex Diseases	
						RB	External	Sunburn	
		*Fragaria vesca* L./AF-91	Budi meva	H	LF	DE	Internal	*Mouth ulcer, *Gum inflammation	1∎, 2●, 3●, 4●, 5●, 6●,7∎, 8●, 9●, 10●, 11●, 12●,13●, 14●, 15●, 16●,17●,18●, 19●, 20●, 21●,22●
					FR	JU	Internal	Anemia, Kidney diseases	
		*Prunus persica* (L.) Batsch./AF-75	Aru	T	LF	JU	Internal	**To kill intestinal worms,** Whooping cough	1●, 2●, 3∎, 4◆, 5●, 6●,7∎,8∎,9∎,10●, 11∎, 12●, 13●, 14●, 15●,16●,17●,18●, 19●, 20●, 21●,22●
						PD	External	Wounds	
					BR	CH	External	*Toothache	
					FL	EX	Internal	Gastrointestinal problems	
		*Pyrus malus* L./AF-98	Saib	T	FR	JU	Internal	**Body weakness,** Joint problems, *Heart disease Hypertension,	1●, 2●, 3∎, 4∎, 5●, 6●,7●, 8●, 9●, 10●, 11●, 12●,13●, 14●,15●, 16●, 17●, 18●, 19●, 20●, 21●,22●
						PA	External	Face spots	
					FL	TE	Internal	*Respiratory and *Nerves disorders	
		*Pyrus pashia* Buch.-Ham.ex D. Don. /AF-85	Tangi	T	FR	ET	Internal	**Dark circles around eyes**	1●, 2●, 3◆, 4◆, 5●, 6●, 7∎, 8●, 9●, 10●, 11●, 12∎, 13●, 14●, 15●, 16●, 17●, 18●, 19●, 20●, 21●, 22●
		*Rosa brunonii* Lindl./AF-103	Jangli Gulab/Chal	S	BA	IN	Internal	*Blood purification	1●, 2●, 3◆, 4◆, 5●, 6∎, 7◆, 8●, 9◆, 10●,11●, 12●, 13●, 14●, 15●, 16●, 17●, 18●, 19●, 20∎, 21●, 22●
					FL	DE	Internal	Constipation	
						PD	External	Skin infection	
		*Rubus fruticosus* L./AF-54	Kanachi	S	FR	EX	Internal	*Tonic	1●, 2∎, 3∎, 4∎, 5∎, 6●, 7∎, 8●, 9●, 10∎, 11●,12●,13∎, 14∎, 15●, 16●, 17∎, 18●, 19●, 20●, 21●, 22●
						ET	Internal	*Sore throat	
					LF	IN	Internal	Diarrhea, *Bleeding	
		*Rubus ellipticus* Sm./AF-52	Akhrayar	S	FR	JU	Internal	Fever, **Cough,** Sore throat	1∎, 2●, 3●, 4●, 5∎, 6●,7●, 8●, 9◆,10●, 11●, 12●,13●, 14●, 15●, 16●, 17●,18●, 19●, 20●, 21●, 22●
					RT	DE	Internal	Fever	
					LB	JU	Internal	*Peptic ulcer	
		*Rubus niveus* Thunb./AF-67	Pahvonny	S	RT	DE	Internal	**Whooping cough,** Dysentery	1●, 2●, 3∎, 4∎, 5●, 6●,7●,8●,9●, 10●, 11●,12●,13●,14●, 15●,16●,17●, 18●, 19●, 20●, 21●, 22●
						EX	External	Wound healing, *Antitumor	
					LF	IN	Internal	*Blood purifier	
44	Rubiaceae	*Rubia cordifolia* L./AF-71	Chero	C	LF	PD	Internal	*Cough	1●, 2●, 3●, 4●, 5●, 6●, 7●, 8●, 9∎, 10●, 11●, 12●,13●, 14●, 15●, 16●, 17●, 18●, 19●, 20●, 21●, 22●
							External	*Broken Bones	
					RT	IN	Internal	*TB, *Lung Cancer, *Nervous disorders, *Gout	
						PA	External	Wounds	
45	Rutaceae	*Zanthoxylum alatum* Roxb./AF-12	Timbar	S	BA	IN	Internal	Stomach disease, To kill intestine worms, Fever	1●, 2●, 3●, 4●, 5●, 6●,7●, 8●, 9∎, 10●, 11●, 12●, 13●, 14●,15●,16●,17●,18●, 19●, 20●,21●,22●
					TW	RB	External	**Toothache**	
					FR	JU	Internal	Indigestion, Cholera	
					SD	EX (Oil)	External	*Antiviral	
						PD	External	Toothache, Gum pain	
46	Salicaceae	*Salix nigra* Marshall./AF-96	Bees	T	BA	PO	External	**To remove swelling**	1●, 2●, 3●, 4●, 5●, 6●, 7●, 8●, 9●, 10●, 11●, 12●, 13●, 14●, 15●, 16●, 17●,18●, 19●, 20●, 21●, 22●
						PD	Internal	Dysentery, Arthritis	
					LF	DE	Internal	To reduce pain, Fever	
47	Sapindaceae	*Aesculus indica* (Wall. ex Cambess.) Hook./AF-5	Banakhori	T	BA	IN	Internal	*Fever	1∎, 2●, 3∎, 4●, 5●, 6∎,7●,8●,9∎, 10●, 11●, 12●, 13●, 14●, 15●, 16●, 17●, 18●, 19●, 20●, 21●,22●
					SD	Oil	External	*Gout disease	
					FR	PD	Internal	*Indigestion	
48	Simaroubaceae	*Ailanthus altissima* (Mill.) Swingle/AF-1	Dravia	T	BA	IN	Internal	Diarrhea, *Dysentery	1●, 2●, 3●, 4●, 5◆, 6●, 7∎, 8●, 9∎, 10●, 11●, 12●, 13●, 14●,15●, 16●, 17∎, 18●, 19●, 20●, 21●,22●
						EX	Internal	*Anemia	
					FR	JU	Internal	*Dysentery, *Bloody stools	
					LE	EX/PD	Internal	*To remove Tapeworms	
49	Solanaceae	*Solanum nigrum* L./AF-109	Kach Mach	H	FR	ET	Internal	**Mouth ulcer**	1●, 2∎, 3●, 4●, 5∎, 6●, 7●, 8●, 9∎, 10∎, 11●,12●,13∎,14∎, 15●, 16∎, 17∎,18∎, 19●, 20●, 21∎, 22∎
					LF	JU	Internal	*Gout, Stomach worm	
						PA	External	Skin disorders	
						CH	External	Mouth Ulcer	
					WP	IN	Internal	Diuretic, Abdominal disorders	
50	Thymelaeaceae	*Daphne papyracea* Wall.ex G. Don. /AF-53	Lokat Patr	S	RT	Extract	Internal	Intestinal complaints	1●, 2●, 3●, 4●, 5●, 6●, 7●,8●,9●, 10●, 11●,12●, 13●, 14●,15●,16●,17●,18●, 19●, 20●, 21●,22●
					LE	Paste	External	Swelling, Tumor	
					ST	Paste	External	**Snake bite**	
		*Wikstroemia canescens* Wall. ex Meisn./AF-117	Chianthi	S	AP	DE	Internal	**Abortifacient**	1●, 2●, 3●, 4●, 5●, 6●, 7●,8●,9●, 10●, 11●,12●, 13●, 14●,15●,16●,17●,18●, 19●, 20●, 21●,22●
51	Urticaceae	*Debregeasia salicifolia* (D. Don) Rendle. /AF-99	Sindwari	S	LE	Powder	External	Skin diseases	1●, 2●, 3∎, 4∎, 5●, 6●,7●, 8●, 9∎, 10●, 11●,12●, 13●, 14●, 15●, 16●,18●, 19●, 20●, 21●,22●
						Infusion	Internal	*Jaundice	
					FR	Juice	Internal	*Bloody diarrhea	
52	Valerianacea	*Valerianella muricata* (Steven ex Roem. & Schult.) W.H. Baxter./AF-47	Cornsalad	H	LF	EX	Internal	**Nerve complaints**	1●, 2●, 3●, 4●, 5●, 6●, 7●, 8●, 9●, 10●, 11●, 12●, 13●, 14●, 15●,16●,17●,18●, 19●, 20●, 22●
53	Verbenaceae	*Verbena officinalis* L./AF-138	Neeli Booti	H	RT	JU	Internal	*Stomachache, *Snake bite	1●, 2∎, 3●, 4●, 5●, 6●,7●, 8●, 9●,10●, 11●, 12●, 13●,14●, 15●, 16●,17●,18●, 19●, 20●,21●, 22●
					WP	DE	Internal	*Dropsy	
					SH	PA	External	*Swollen gums	
54	Violaceae	*Viola canescens* Wall. /AF-81	Banafsha	H	WP	JU	Internal	Antipyretic, *High Blood pressure, Asthma, **Cough,** *Flue, *Eye diseases, Stomachache, Liver disease	1●, 2●, 3∎, 4∎, 5∎, 6●,7●, 8∎, 9∎, 10●, 11●, 12●, 13∎, 14●, 15●, 16●,17●, 18●, 19●, 20●, 21●,22∎
					FL	JU	Internal	Cough, Insomnia	
					LF	JU	Internal	Jaundice, Cough	
55	Vitaceae	*Vitis jacquemontii* R. Parker./AF-24	Dakh/Dalore/Jungli Angoor	C	FR	ET	Internal	Tonic, **Constipation,** Laxative	1●, 2●, 3●, 4●, 5●, 6●, 7●, 8●, 9●,10●, 11●,12●, 13●, 14●, 15●, 16●,17●,18●, 19●, 20●, 21●,22●
					ST	JU	Internal	Internal fever	

Habit: *H*, herbs, *S* shrubs, *T* trees, *C* climber, *E* epiphyte; 2. Part(s) used: *LE* leaf, *FR* fruit, *RT* Root, *ST* stem, *AP* aerial Parts, *ND* needles, *WP* whole Plant, *FD* fronds, *SD* Seed, *FL* flower, *BA* bark, *BL* bulb, *RH* rhizome, *IN* inflorescence, *PL* pollen, *TW* twig, *SH* shoot, *LX* latex, *LB* leaf bud, *GL* galls, *BR* branches, *FP* floral parts, *RS* resin; 3. Method of preparation: *PD* powder, *DE* decoction, *EX* extract, *PA* paste, *JU* juice, *PO* poultice, *IN* infusion, *HR* hot rubbing, *CH* chewed, *VG* vegetable, *TE* tea, *RB* rubbing, *ET* eaten, *CK* cooked, *HB* hot beverage. (◆) = plants with similar use(s); (∎) = plants with dissimilar use (s); (●) = plants not reported in a previous study; Condition/ailment written in bold indicate the most preferred use for a given plant; *Plant uses, which are not reported in a previous study. 1: Ahmad et al. [20]; 2: Hussain et al. [60] 3: Shaheen et al. [61]; 4: Amjad et al. [59]; 5: Ajaib et al. [62]; 6: Safeer et al. [63]; 7: Shabir et al. [64]; 8: Ahmad and Habib, [65]; 9: Qaseem et al. [66]; 10: Khan et al. [67]; 11: Wali et al. [68]; 12: Ijaz et al. [69]; 13: Hussain et al. [70]; 14: Aziz et al. [71]; 15: Ahmad et al. [39]; 16: Aziz et al. [50]; 17: Gulzar et al. [45]; 18: Umair et al. [36]; 19: Zahoor et al. [72]; 20: Kayani et al. [38]; 21; Umair et al. [73]; 22: Fatima et al. [74]

### Plant part(s) used

**Fig. 5:**
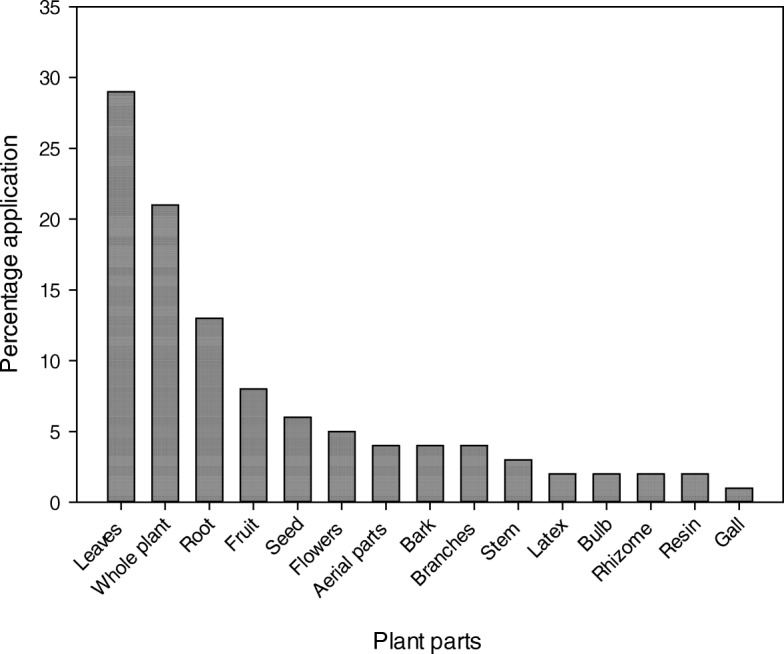
Plant parts used in herbal recipes

Data presented in Fig. 5 revealed that local inhabitants of the study area use 15 different parts of plants in making recipes to treat various diseases. Among these, leaves were the most abundantly utilized plant parts with percentage contribution of 29%, followed by whole plants (21%) and root (13%), fruit (8%), seed (6%), and flowers (5%) contribution, whereas the use of aerial parts, bark, branches, stem, and latex etc. were less than 5%. Abundant availability and easy collection or harvesting of leaves make them highly utilized plant parts [4, 61, 72, 83]. Moreover, leaves also contain a high concentration of health-beneficial secondary metabolites, phytochemicals, and essential oils, which contribute significantly to phytotherapy or treatment of various health disorders [15, 75, 84]. Likewise, roots are storage parts of plant species also rich in bioactive constituents compared to other parts [4, 85, 86], which therefore possess more health-beneficial properties if collected in the proper time. However, previous studies revealed that majority of the researchers supported the use of leaves than roots, because eradication of roots may lead to serious conservation threats to various plant species particularly those which are highly utilized [60, 87, 88]. Moreover, it is not an easy job to collect the roots of woody and deep-rooted plants [39]. The frequent utilization of the whole plant in preparation of herbal remedies confirmed the abundant utilization of herbs in the investigated area as the whole plant can be used only in the case of herbs.

### Herbal preparation and administration

**Fig. 6:**
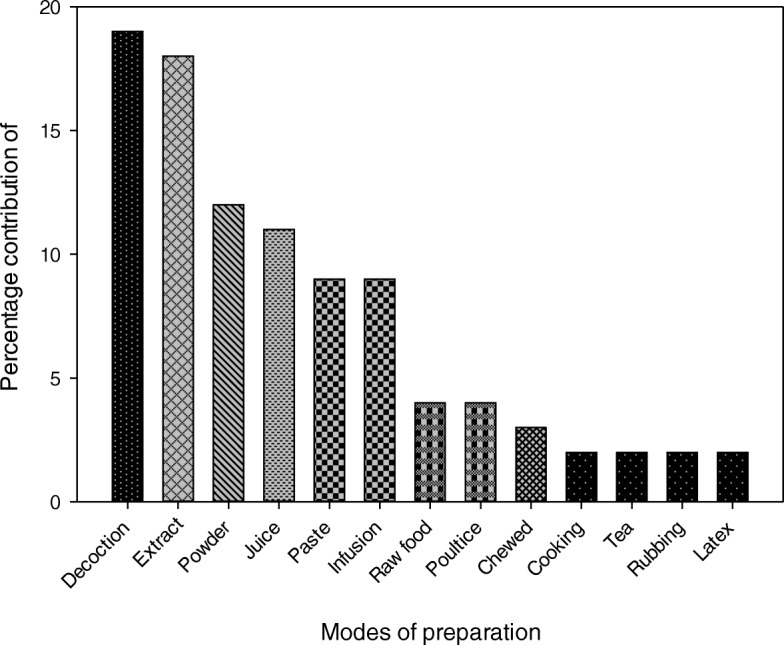
Methods of preparation of herbal recipes

Decoction was the widespread used method in the study area for herbal preparation with percentage contribution of 19%, followed by extract, powder, and juice used in 18, 12, and 11% preparations of traditional recipes, respectively (Fig. 6). The frequent use of decoction had also been reported previously [36, 39, 53, 73, 81, 89, 90]. This confirms that making decoction is a very simple and easy way used for herbal preparation with more health benefits [91]. In decoction form, the efficacy of herbal remedies increases due to the maximum extraction of health-beneficial secondary metabolites and other bioactive compounds, which is accelerated on heating [92]. Taste of medicines can be adjusted by adding honey or sugar to make it more pleasant [39, 93]. Inhabitants of the study area use 63% of the herbal preparations as oral intake, whereas rest 37% were applied topically. These results were analogous to previous reports [36, 67, 68, 72, 94, 95]. Poultice, rubbing, and paste were common topical methods as reported in previous studies [51, 96]. In oral mode of administration, plant materials were mainly ingested as a decoction or in powder form with water, milk, or honey. These results are analogous to the previous findings [49, 97]. Oral intake of herbal preparation is usually effective for the treatment of internal diseases, while for external diseases, i.e., skin infections, joint pain, hemorrhoid, and stings, were treated by topical application of the drug. These observations were in agreement with previous reports [98].

### Informant consensus factor

**Fig. 7:**
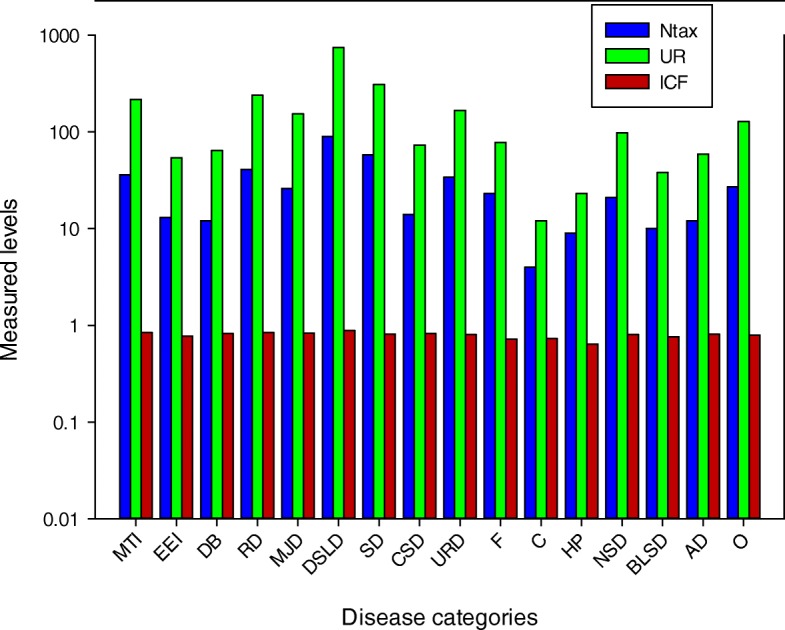
Informant consensus factor of diseases with the use reports and the total number of species used. Ntax, total species used by all the informants for a group of ailment; Nur, total number of use reports in each group of disease; ICF, informant consensus factor; *MTI* mouth-throat infections; EEI, eye and ear infections; DB, diabetes; RD, respiratory disorders; MID, muscular and Joint disorders; DSLD, digestive system and Liver diseases; SD, skin diseases; CSD, circulatory system diseases; URD, urinary and reproductive diseases; F, fever; C, cancer; HP, hair problems; NSD, nervous system disorders; BLSD, blood and lymphatic system diseases; AD, antidote; O, others

Different diseases reported from Dhirkot were classified into 16 categories to develop the consensus of informants on medicinal plants following WHO’s international categorization of ailments [99]. As mentioned in Fig. 7, informant consensus factor (ICF) values ranged from 0.64 to 0.88 with the highest level of 0.88 for gastrointestinal disorders and liver diseases. Prevalence of gastrointestinal disorders is mainly attributed to poor hygiene conditions, inadequate supply of pure drinking water, and consumption of contaminated food [100, 101]. *Allium cepa*, *Allium sativum*, *Mentha arvensis*, *Mentha longifolia*, *Viola canescens*, *Vitis jacquemontii*, *and Zanthoxylum alatum* were among the most frequently utilized plant species to treat digestive system and liver diseases in the study area. Likewise, more consumption of a high-calorie fatty diet in the local communities and changing lifestyle could be the possible reasons of liver diseases in the study area. Our data revealed that around 90 plant species with 743 used reports were used to treat liver disorders. The plant species used to treat digestive and liver diseases have been reported as a rich source of flavonoids, toxol, vitamins, and essential oils along with other bioactive phytochemicals [102, 103]. Additionally, inhabitants of the study area have traditional knowledge due to more interaction with these plant species, particularly used to treat digestive and liver disorders. Comparative assessment with previous studies exposed that many workers have also reported the highest ICF for digestive problems [61, 70, 71, 81, 104, 105].

The second highest ICF value viz. 0.84 was calculated for respiratory tract and throat diseases. Different factors such as sudden changes in weather, poor hygiene conditions, a high proportion of cold, moisture, germs, and spores may cause abnormalities in the respiratory track [51, 81]. *Swertia cordata*, *Trifolium pretense*, *Viola canescens*, *Elaeagnus umbellate*, and *Achyranthes aspera* were among the commonly utilized plant species for the treatment of respiratory infections. In our study, the high ICF value for skin disease might be due to the fact that local inhabitants residing in mountains at a higher altitude are more exposed to UV radiations along with other pathogenic attacks that may lead to chronic skin diseases and infections [106–108]. The most common species used to treat skin diseases were *Adiantum caudatum*, *Ajuga bracteosa*, *Achillea millefolium*, *Berberis lycium*, *Cedrus deodara*, *Cynodon dactylon*, *Daphne papyracea*, *Debregeasia salicifolia*, *Ficus carica*, *Ficus palmate*, and *Gerbera gossypina*.

Muscular and joint diseases are also common in the study area, which might be due to stress, minor injuries, and unhealthy food. Inhabitants of the study area use *Ricinus communis*, *Rubia cordifolia*, *Salix nigra*, *Sarcococca saligna*, and *Sigesbeckia orientalis* to treat joint and muscular problems. Urinary and reproductive system diseases are also common due to the unawareness and excessive use of medications. Moreover, abnormality in hormonal production, malnutrition, and environmental factor may cause reproductive disorders. The inhabitants of the study area use *Saccharum spontaneum*, *Sarcococca saligna*, *Sorghum halepense*, *Trifolium pretense*, *Wikstroemia canescens*, *Eriophorum comosum* to treat reproductive disorders. The lowest ICF value was calculated for hair problems (0.64) and 9 species including *Allium cepa*, *Allium sativum*, *Melia azadarach*, *Olea ferruginea*, and *Ricinus communis* were used to treat this disease with 23 use reports.

### Relative importance

RI of plant species is a useful parameter to measure their adaptability. Data presented in Table 3, indicates that RI values of the reported species varied from 12.14–92.90, which were comparable with previous reports [80]. The highest RI value was calculated for *Viola canescens* (92.86), followed by *Chenopodium ambrosioides*, *Pinus roxburghii*, *Conyza Canadensis*, *Jasminum grandiflorum* (90.00, 82.86, 77.86, and 77.86, respectfully), whereas *Pyrus malus*, *Galinsoga parviflora,* and *Hydrocotyle* spp. have the same RI value (70.71 each). Plants with the highest RI indicate that they are primarily used by the inhabitants of the area and possess strong pharmacological properties [59] and their importance increases when it is used to cure more infirmities [109].

**Table 3:** Quantitative analysis of ethnobotanical data

Sr.#	Scientific name	Rel. PH	Rel. BS	RI	FC	RFC	UV
1	*Acacia nilotica*	0.50	0.57	53.57	31.0	0.42	0.70
2	*Achillea millefolium*	0.60	0.57	58.6	57.0	0.77	0.96
3	*Achyranthes aspera*	0.40	0.57	48.6	63.0	0.85	1.30
4	*Adiantum caudatum*	0.50	0.57	53.6	29.0	0.40	0.73
5	*Adiantum tenerum*	0.50	0.57	53.6	22.0	0.30	0.65
6	*Aesculus indica*	0.30	0.43	36.4	19.0	0.26	0.54
7	*Ailanthus altissima*	0.50	0.29	39.3	21.0	0.30	0.42
8	*Ajuga bracteosa*	0.40	0.29	34.3	54.0	0.73	0.93
9	*Ajuga parviflora*	0.30	0.43	36.4	28.0	0.38	0.55
10	*Allium cepa*	0.40	0.43	41.4	49.0	0.66	0.88
11	*Allium sativum*	0.50	0.71	60.7	51.0	0.70	0.82
12	*Amaranthus viridis*	0.30	0.43	36.4	30.0	0.40	0.61
13	*Androsace rotundifolia*	0.30	0.43	36.4	39.0	0.53	0.74
14	*Arthraxon prionodes*	0.20	0.29	24.3	11.0	0.15	0.20
15	*Aristida cyanantha*	0.30	0.29	29.3	20.0	0.30	0.35
16	*Artemisia vulgaris*	0.20	0.29	24.3	53.0	0.72	0.83
17	*Asplenium dalhousiae*	0.40	0.43	41.4	29.0	0.40	0.54
18	*Berberis lycium*	0.50	0.71	60.7	64.0	0.86	1.30
19	*Bidens biternata*	0.20	0.14	17.1	39.0	0.53	0.65
20	*Bromus catharticus*	0.20	0.29	24.3	10.0	0.13	0.22
21	*Campanula pallida*	0.20	0.14	17.3	14.0	0.19	0.26
22	*Cannabis sativa*	0.20	0.29	24.3	24.0	0.32	0.55
23	*Capsella bursa-pastoris*	0.30	0.43	36.4	33.0	0.44	0.62
24	*Carpesium cernuum*	0.60	0.57	58.6	23.0	0.31	0.42
25	*Cedrus deodara*	0.60	0.57	58.6	17.0	0.23	0.54
26	*Chenopodium ambrosioides*	0.80	1.00	90.0	36.0	0.50	0.72
27	*Chrysopogon gryllus*	0.10	0.14	12.1	8.0	0.11	0.11
28	*Cichorium intybus*	0.70	0.43	56.4	39.0	0.53	0.23
29	*Cirsium vulgare*	0.30	0.43	36.4	19.0	0.26	0.46
30	*Clematis grata*	0.20	0.29	24.3	23.0	0.39	0.40
31	*Convolvulus arvensis*	0.20	0.29	24.3	15.0	0.20	0.31
32	*Conyza canadensis*	0.70	0.86	77.9	43.0	0.60	0.70
33	*Cymbopogon martini*	0.50	0.43	46.4	13.0	0.20	0.30
34	*Cynodon dactylon*	0.50	0.57	53.6	37.0	0.50	0.62
35	*Cynoglossum lanceolatum*	0.50	0.29	39.3	42.0	0.60	0.76
36	*Cyperus serotinus*	0.20	0.29	24.3	11.0	0.15	0.20
37	*Dactylis glomerata*	0.50	0.57	53.6	23.0	0.31	0.40
38	*Daphne papyracea*	0.40	0.57	48.6	16.0	0.22	0.32
39	*Debregeasia salicifolia*	0.30	0.29	29.3	20.0	0.30	0.44
40	*Desmodium elegans*	0.60	0.71	65.7	26.0	0.35	0.67
41	*Dichanthium annulatum*	0.30	0.43	36.4	12.0	0.20	0.30
42	*Dicliptera roxburghiana*	0.30	0.43	36.4	32.0	0.43	0.52
43	*Diospyros lotus*	0.30	0.43	41.4	41.0	0.55	0.72
44	*Dryopteris filix-mas*	0.50	0.43	46.4	25.0	0.34	0.46
45	*Duchesnea indica*	0.30	0.43	36.4	29.0	0.40	0.54
46	*Elaeagnus umbellata*	0.40	0.29	34.3	44.0	0.60	0.80
47	*Eleusine indica*	0.30	0.43	36.4	10.0	0.13	0.20
48	*Eriophorum comosum*	0.20	0.29	24.3	8.0	0.10	0.14
49	*Euphorbia indica*	0.50	0.43	46.4	26.0	0.35	0.63
50	*Euphorbia prostrata*	0.40	0.29	34.3	19.0	0.26	0.50
51	*Ficus carica*	0.60	0.71	65.7	48.0	0.65	0.78
52	*Ficus palmata*	0.50	0.43	46.4	53.0	0.72	0.85
53	*Fragaria nubicola*	0.40	0.57	48.6	27.0	0.36	0.53
54	*Fragaria vesca*	0.40	0.43	41.4	33.0	0.44	0.55
55	*Galinsoga parviflora*	0.70	0.71	70.7	22.0	0.30	0.61
56	*Gentianodes olivieri*	0.30	0.43	36.4	12.0	0.16	0.23
57	*Gerbera gossypina*	0.30	0.29	29.3	29.0	0.40	0.63
58	*Hedera nepalensis*	0.30	0.29	29.3	32.0	0.43	0.51
59	*Hydrocotyle* spp.	0.70	0.71	70.7	26.0	0.35	0.55
60	*Hypericum perforatum*	0.70	0.43	56.4	37.0	0.50	0.62
61	*Impatiens edgeworthii*	0.30	0.43	36.4	11.0	0.15	0.34
62	*Impatiens glandulifera*	0.30	0.43	36.4	19.0	0.26	0.42
63	*Indigofera heterantha*	0.20	0.29	24.3	32.0	0.43	0.55
64	*Inula* spp.	0.40	0.57	48.6	21.0	0.29	0.46
65	*Ipomoea purpurea*	0.60	0.57	58.6	34.0	0.46	0.55
66	*Isodon rugosus*	0.50	0.29	39.3	40.0	0.54	0.70
67	*Jasminum grandiflorum*	0.70	0.86	77.9	54.0	0.73	0.82
68	*Justicia vahlii*	0.10	0.14	12.1	9.0	0.12	0.15
69	*Lespedeza juncea*	0.40	0.43	41.4	22.0	0.30	0.40
70	*Machilus odoratissimus*	0.30	0.43	36.4	16.0	0.23	0.34
71	*Malva parviflora*	0.40	0.57	48.6	44.0	0.60	0.76
72	*Matricaria matricarioides*	0.50	0.43	46.4	23.0	0.31	0.40
73	*Medicago lupulina*	0.20	0.29	24.3	34.0	0.46	0.54
74	*Melia azedarach*	0.50	0.71	60.7	50.0	0.70	0.76
75	*Mentha arvensis*	0.50	0.14	32.1	65.0	0.88	0.96
76	*Mentha longifolia*	0.40	0.29	34.3	53.0	0.72	0.82
77	*Micromeria biflora*	0.30	0.43	36.4	20.0	0.30	0.35
78	*Morus alba*	0.30	0.43	36.4	38.0	0.51	0.62
79	*Myriactis wallichii*	0.10	0.14	12.1	11.0	0.15	0.20
80	*Myrsine africana*	0.40	0.43	41.4	53.0	0.72	0.82
81	*Nepeta laevigata*	0.30	0.43	36.4	20.0	0.30	0.31
82	*Nerium oleander*	0.30	0.43	36.4	43.0	0.60	0.81
83	*Oenothera rosea*	0.20	0.29	24.3	36.0	0.50	0.60
84	*Olea ferruginea*	0.40	0.57	48.6	52.0	0.76	0.82
85	*Onychium japonicum*	0.40	0.43	41.4	18.0	0.24	0.42
86	*Oplismenus compositus*	0.10	0.14	12.1	15.0	0.20	0.26
87	*Origanum vulgare*	0.40	0.57	48.6	28.0	0.40	0.50
88	*Oxalis corniculata*	0.40	0.43	41.4	48.0	0.65	0.74
89	*Parthenium hysterophorus*	0.60	0.71	65.7	37.0	0.50	0.61
90	*Pennisetum orientale*	0.10	0.14	12.1	17.0	0.23	0.30
91	*Persicaria capitata*	0.60	0.71	65.7	21.0	0.30	0.40
92	*Phagnalon rupestre*	0.40	0.43	41.4	28.0	0.38	0.44
93	*Pinus roxburghii*	0.80	0.86	82.9	57.0	0.80	0.90
94	*Pinus wallichina*	0.50	0.57	53.6	51.0	0.70	0.82
95	*Plantago lanceolata*	0.40	0.29	34.3	43.0	0.60	0.76
96	*Planatus orientalis*	0.50	0.57	53.6	30.0	0.40	0.55
97	*Plectranthus rugosus*	0.20	0.29	24.3	37.0	0.50	0.62
98	*Polygonum hydropiper*	0.60	0.71	65.7	29.0	0.40	0.50
99	*Prenanthes brunoniana*	0.20	0.14	17.1	19.1	0.26	0.32
100	*Prunella vulgaris*	0.40	0.57	48.6	48.0	0.65	0.88
101	*Prunus persica*	0.50	0.57	53.6	57.0	0.77	0.89
102	*Pteracanthus urticifolius*	0.50	0.71	60.7	26.0	0.35	0.45
103	*Pteris cretica*	0.20	0.29	24.3	8.0	0.10	0.15
104	*Pteris vittata*	0.40	0.43	41.4	13.0	0.17	0.26
105	*Punica granatum*	0.40	0.43	41.4	55.0	0.74	0.89
106	*Pyrus malus*	0.70	0.86	77.9	58.0	0.80	0.87
107	*Pyrus pashia*	0.20	0.29	24.3	53.0	0.72	0.90
108	*Quercus incana*	0.50	0.71	60.7	55.0	0.74	0.86
109	*Ranunculus arvensis*	0.50	0.71	60.7	21.0	0.28	0.34
110	*Ranunculus muricatus*	0.30	0.43	36.4	12.0	0.22	0.18
111	*Ricinus communis*	0.60	0.71	65.7	36.0	0.49	0.65
112	*Rosa brunonii*	0.30	0.43	36.4	45.0	0.61	0.77
113	*Rubia cordifolia*	0.60	0.71	65.7	39.0	0.53	0.62
114	*Rubus fruticosus*	0.40	0.57	48.6	50.0	0.68	0.84
115	*Rubus ellipticus*	0.40	0.57	48.6	42.0	0.56	0.62
116	*Rubus niveus*	0.50	0.71	60.7	28.0	0.38	0.52
117	*Rumex dentatus*	0.30	0.14	22.1	45.0	0.61	0.62
118	*Rumex hastatus*	0.20	0.29	24.3	40.0	0.54	0.69
119	*Saccharum spontaneum*	0.60	0.57	58.6	24.0	0.32	0.43
120	*Salix nigra*	0.50	0.71	60.7	30.0	0.40	0.49
121	*Salvia lanata*	0.50	0.43	46.4	21.0	0.30	0.44
122	*Sarcococca saligna*	0.30	0.43	36.4	18.0	0.24	0.31
123	*Setaria viridis*	0.30	0.43	36.4	15.0	0.20	0.26
124	*Sigesbeckia orientalis*	0.60	0.57	58.6	33.0	0.44	0.54
125	*Solanum nigrum*	0.60	0.71	65.7	54.0	0.73	0.85
126	*Sonchus arvensis*	0.40	0.43	41.4	23.0	0.31	0.38
127	*Sonchus oleracus*	0.60	0.43	51.4	29.0	0.40	0.44
128	*Sorghum halepense*	0.50	0.57	53.6	12.0	0.16	0.20
129	*Swertia cordata*	0.50	0.71	60.7	49.0	0.70	0.84
130	*Swertia paniculata*	0.30	0.43	36.4	24.0	0.32	0.42
131	*Tagetes minuta*	0.40	0.43	41.4	40.0	0.54	0.78
132	*Taraxacum officinale*	0.50	0.29	39.3	63.0	0.85	0.86
133	*Trifolium pratense*	0.50	0.57	53.6	36.0	0.49	0.57
134	*Valerianella muricata*	0.10	0.14	12.1	11.0	0.15	0.17
135	*Verbena officinalis*	0.40	0.57	48.6	27.0	0.36	0.42
136	*Viburnum grandiflorum*	0.20	0.14	17.1	22.0	0.30	0.34
137	*Viola canescens*	1.00	0.86	92.9	68.0	0.92	1.70
138	*Vitis jacquemontii*	0.40	0.43	41.4	16.0	0.22	0.31
139	*Wikstroemia canescens*	0.10	0.14	12.1	9.0	0.12	0.15
140	*Zanthoxylum alatum*	0.80	0.57	68.6	61.0	0.82	0.89

*Rel. PH* relative number of pharmacological properties attributed to a single plant, *Rel. BS* relative number of body systems treated by a single species, *RI* relative importance, *FC* frequency of citation, *RFC* relative frequency of citation, *UV* use value

### Relative frequency of citation

Relative frequency of citation (RFC) indicates the native importance of each plant species with respect to informants who reported the uses of these species [[5]. The RFC value of reported species ranged from 0.1 to 0.92 (Table 3). The highest RFC was calculated for *Viola canescens* (0.92) and, subsequently, *Mentha arvensis* (0.88), *Berberis lycium* (0.86), *Achyranthes aspera* (0.85), *Taraxacum oficinale* (0.85), *Zanthoxylum alatum* (0.82), *Pinus roxburghii* (0.80), *Pyrus malus* (0.80), *Achillea millefolium* (0.77), and *Prunus persica* (0.77). The high RFC value of these species indicates that inhabitants of the study area have a close association with these plant species and frequently use them to treat various diseases. The RFC data may contribute significantly to understand the importance of a plant species within an area, to conserve plant species having maximum RFC, and for biological, pharmacological, and phytochemical screening of such species. The high RFC of *Viola canescens* indicates that this species is commonly utilized by local communities to treat various health disorders. This leads to overexploitation of this species in the study area indicating a high conservation threat and may lead to extension into the future if not conserved immediately. Likewise, some plants having high RFC are rare in the study area and vice versa. For example, *Rauvolfia serpentia* is a rare plant in the study area but had a high FC (FC-43) value.

### Use value

The use value (UV) index was used to measure the ethnomedicinal uses associated with documented medicinal plant species and is ranged from 0.11–1.7 (Table 3). The highest UV was reported for *Viola canescens* (1.7), followed by *Achyranthes aspera* (1.3), *Achillea millefolium* (0.96), *Mentha arvensis* (0.96), *Ajuga bracteosa* (0.93), *Pinus roxburghii* (0.9), *Pyrus pashia* (0.90), *Prunus persica* (0.89), *Punica granatum* (0.89) *Allium cepa* (0.88), and *Prunella vulgaris* (0.88). The high usage of the reported species indicates a strong association and dependence of local communities on surrounding flora, specifically for the treatment of various diseases and as food and livelihoods [51]. Moreover, the plant species which are used excessively are assumed to be biologically more active; therefore these should be subjected to phytochemical and pharmacological screening to increase sustainable utilization and conservation of plant resources [110].

### Fidelity level

**Fig. 8:**
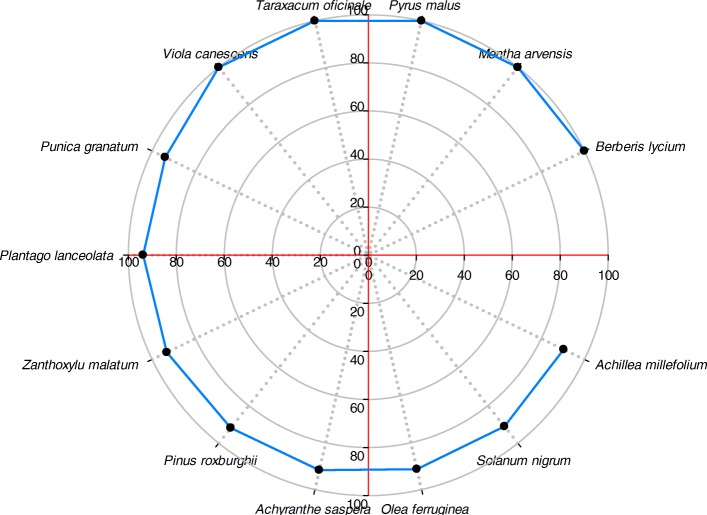
Top-ranked plant species with above 90% fidelity level

FL identifies the most preferred plant species used by traditional healers to cure various diseases and shows the proportion of informants reporting the use of specific plant species. The FL level of reported species was ranged from 15.8–100%. Figure 8 shows some top-ranked species with FL above 90%. Among these, five plant species which include *Berberis lyceum*, *Mentha arvensis*, *Pyrus malus*, *Taraxacum officinale*, and *Viola canescens* (for wound healing, to treat gastrointestinal disorders, body weakness, diabetes, and cough, respectively) have 100% fidelity level, whereas *Morus alba* had the lowest FL (15.8%) and was used to treat body weakness. These findings elucidate the dominance of specific ailments in the area that are cured with different plant species, particularly having high FL [81]. Plant species having high FL values are extensively used in the area compared to those with less FL values and similar findings have already been reported [35]. These plants are used to cure different ailments since ancient times in combination with other plants or ingredients and could be considered as model plants for pharmacological screening [38]. Despite the fact that modern health facilities are accessible in the study area, local communities especially in the mountainous parts of this region still rely on medicinal plants and possess significant traditional knowledge on plant resource utilization.

### Novel uses

The comparison of indigenous knowledge on medicinal plants is helpful to determine the difference between region arising due to ecological [111], historical [112], organolep,tic and phytochemical differences [71, 113]. The Jaccard index (JI) is a quantitative index used to compare the ethnobotanical data with previous reports, specifically from adjoining areas. In this study, the data was compared with 22 previously published articles. The similarity percentage with the allied area ranges from 2.08–14.9, whereas our findings were dissimilar up to 41.8 from previous data (Table 4). The highest JI value (48.4) was with data reported previous [64] from Devi Galli Azad Kashmir, Pakistan. This similarity was due to the fact that both areas have the same type of vegetation and geography along with a similarity in culture and cross-cultural exchange of traditional knowledge among communities. Conversely, our data depicted the lowest similarity (JI = 2.08) with reported ethnomedicinal uses of plant species from Central Punjab, Pakistan [7]. These variations might be due to cultural diversity, geo-climatic conditions, habitat structure, and change on vegetation types of bath areas. More specifically, the origin and culture of local communities have a significant influence on ethno-ecological knowledge.

**Table 4:** Jaccard index comparing the present study with previous articles

Sr. no.	Study area	SY	Np	NRPs	NPSU	NPDU	TSCBA.	SEAA50-18	SESA140-18	PPSU 3/50 × 100	PPDU 15/50 × 100	JI	C
A	Comparison with articles from AJK												
1	Neelum (AJK), Pakistan	2017	20	50	3	15	18	32	122	6.00	30.0	13.2	[20]
2	Bhimber (AJK), Pakistan	2013		97	5	20	25	72	115	5.15	20.62	15.4	[60]
3	Rawalakot, (AJK), Pakistan	2017	64	136	16	27	43	93	97	11.8	19.85	29.3	[61]
4	Toli Peer National Park, (AJK), Pakistan	2017	64	121	18	24	42	79	98	14.9	19.8	31.1	[59]
5	Darguti, Tehsil khuiratta, AJK,Pakistan	2015		100	6	28	34	66	106	6.00	28	24.6	[62]
6	Bagh, (AJK), Pakistan	2017		34	3	13	16	18	124	8.8	38.2	12.7	[63]
7	Devi Galli Azad Kashmir	2017	135	98	6	41	47	51	93	6.12	41.8	48.4	[64]
8	Neelum, (AJK), Pakistan	2014	100	59	2	19	21	38	119	3.4	32.2	15.4	[65]
9	District Kotli, (AJK),Pakistan	2019	112	80	7	21	28	52	112	8.75	26.25	20.6	[66]
B	Comparison with articles from Northern Pakistan												
10	Dir Lower, Pakistan	2018	87	50	2	20	22	28	118	4	40	17.7	[67]
11	Gilgit Baltistan, Pakistan	2019	146	90	2	14	16	74	124	2.2	15.5	8.80	[68]
12	Sarban Hills, Abbottabad, Pakistan	2016	134	74	4	17	21	53	119	5.4	22.9	13.9	[69]
13	Northern Pakistani Afghan borders	2018	108	92	2	23	25	67	115	2.8	25	16.0	[70]
14	Bajaur Agency, Pakistan	2017	108	79	5	18	23	55	116	6.33	22.8	15.5	[71]
15	Chail Valley, District Swat, Pakistan	2014	142	50	7	10	17	33	123	14	20	12.2	[39]
16	South Waziristan agency, Pakistan	2016	113	82	4	17	21	61	119	4.88	20.7	13.2	[50]
17	Malakand, KPK, Pakistan	2019		50	3	14	17	33	123	6	28	12.2	[45]
C	Comparison with articles from whole Pakistan												
18	Hafizabad district, Punjab, Pakistan	2107	166	85	7	11	18	67	122	8.2	12.9	10.5	[36]
19	District Sheikupura, Pakistan	2017	400	96	2	13	15	81	125	2.08	13.54	7.85	[72]
20	Alpine and Sub-alpine regions of Pakistan	2015	290	125	3	12	15	110	125	2.4	9.6	6.80	[38]
21	Chenab riverine, Punjab province Pakistan	2019	321	129	7	13	20	109	120	5.4	10.1	9.60	[73]
22	Central Punjab-Pakistan	2017	197	72	2	7	9	63	131	2.8	9.7	4.90	[74]

*SY* study year, *Np* number of participants, *NRPs* number of reported plant species, *NPSU* number of planst with similar uses, *NPDU* number of plants with different uses, *TSCBA* total species common in both area, *SEAA* species enlicted in aligned areas, *SESA* species enlisted only in study area, *PPSU* percentage of plant with similar uses, *PPDU* percentage of plant with different uses, *JI* Jaccard index, *C* citation

Comparative analysis of present findings with reported literature revealed some new uses of plant species, which have rarely been documented so far from this region, such as the stem ash of *A. nilotica* is used to treat eye infections. Leaves of *A. bracteosa*, *A. rotundifolia*, *B. lyceum*, *I. rugosus*, *P. roxburghii*, and *T. officinale* are used to cure stomach disorders, menstrual problems, and flu and to heal wounds in the form of different formulations (decoction, extract, paste, and powder). Likewise, inhabitants of the study area use fruits of *F. nubicola*, *M. azedarach*, *M. africana*, *O. ferruginea*, and *S. nigrum* for the treatment of diabetes and mouth infections, to remove intestinal worms, and for hair growth (Table 2). Consequently, documenting and comparing such information reflects the considerable intensity of knowledge among local communities, which can provide a novel source of remedial preparation [114] and indicates the high degree of ethnomedicinal novelty in the study area [20, 36].

## Conclusions

Due to its unique geography and diverse climatic conditions, Dhirkot and its allied areas harbor rich botanical and cultural diversity. Though inhabitants of this area have a strong association with surrounding flora and fauna, ethnomedicinal knowledge is at an extreme risk of extinction as it is mainly restricted to traditional healers, midwives, and older people. Consequently, there is a dire need to avoid the extinction of this ethnobotanical heritage that could be attained by the involvement of concerned authorities, conservation managers, and academia. Furthermore, high-value medicinal plant species of this area not only could contribute significantly in the livelihood of the future generations, particularly of this region, but also be a rich source of biomass supply for pharmaceutical industries.

## Data Availability

All data have already been included in the manuscript.
